# Domain-Selected Spin Projection and Orbital-Filling Softening of Charge Plasmons in a Model Altermagnetic RbV_2_Te_2_O Coulomb Bilayer

**DOI:** 10.3390/nano16211430

**Published:** 2026-11-05

**Authors:** Shun Zhou, Qi Zhang, Fangwei Han

**Affiliations:** 1 School of Medical Information Engineering, Jining Medical University, Jining 272067, China; 2 Robinson Research Institute, Victoria University of Wellington, Lower Hutt 5046, New Zealand

**Keywords:** altermagnet, Coulomb bilayer, plasmon, random-phase approximation, hBN

## Abstract

Altermagnets combine compensated magnetic order with momentum-dependent spin splitting, offering spin-polarized states without stray fields. Plasmons in altermagnetic metals can carry a spin signal, but its distribution among the modes of coupled layers is unknown. Here, we study a Coulomb-coupled bilayer of the d-wave altermagnet ${\mathrm{RbV}}_{2}$${\mathrm{Te}}_{2}$O, using a representative four-band tight-binding model with spin–orbit coupling and the random-phase approximation with density-channel Coulomb interaction. We find that, at equal filling in a symmetric environment, switching the relative magnetic domain leaves the charge spectra unchanged but exchanges the total-spin weight between the acoustic (187.4 meV) and optical (413.1 meV) plasmons at *q* = 0.2 ${\mathrm{nm}}^{-1}$. A missing spin peak arises from interlayer cancelation, which the layer-difference spin reveals, or from a symmetry node along the diagonal, where both spin channels vanish. Raising the chemical potential from 0 to 0.2 eV softens the acoustic plasmon by 15% and raises the relative spin amplitude, as the *xy* orbital loses low-energy current weight. With a dynamic hexagonal boron nitride (hBN) spacer, the plasmons move to 214.0 and 409.3 meV; the selection survives, and only about 2% leaks into the dark branch. Charge spectra are domain-independent, so testing requires a spin-sensitive probe.

## 1. Introduction

Antiferromagnets are attractive for nanoscale spintronics because their compensated magnetic order produces no stray field, so that neighboring units do not disturb each other [1]. Altermagnets extend this advantage. Their two spin species are connected by a crystal rotation rather than by a translation or an inversion, so the order is compensated while the bands still show a momentum-dependent, non-relativistic spin splitting that alternates with the direction in momentum space [2,3], as resolved by photoemission in MnTe [4]. Among the metallic candidates, the layered compound ${\mathrm{Rb}}_{1-\delta }$$V_{2}$${\mathrm{Te}}_{2}$O stands out. It is a room-temperature d-wave altermagnet whose angle-resolved spectra show crystal-symmetry-paired spin–valley locking [5], and a G-type antiferromagnetic order has been reported for the same family [6]. Its metallic character provides itinerant carriers with tunable density, and its layered structure makes few-layer stacks natural candidates for devices. A four-band tight-binding model that keeps the orbital and spin character of the low-energy states, with parameters fitted to first-principles bands of ${\mathrm{RbV}}_{2}$${\mathrm{Te}}_{2}$O, is now available [7], which allows the collective electronic response of this altermagnet to be studied at the model level. Such a study bears on spin-selective nanophotonic and spintronic elements built from ${\mathrm{Rb}}_{1-\delta }$$V_{2}$${\mathrm{Te}}_{2}$O.

Plasmons are a natural probe of this response. In two-dimensional conductors, they confine the electromagnetic field to a small fraction of the free-space wavelength, and their energies can be tuned through the carrier density and the dielectric environment [8,9]. When two layers are coupled by the Coulomb interaction, the plasmons split into an in-phase optical branch and an out-of-phase acoustic branch [10,11]. These names follow the terminology of coupled-layer plasmons [10,11]: they describe the relative phase of the charge oscillations in the two layers and have no relation to acoustic or optical phonons. We also call the two branches layer-odd (acoustic) and layer-even (optical). Acoustic plasmons have been imaged in graphene by photocurrent and near-field nanoscopy [12,13] and used as resonators for the infrared spectroscopy of ultrathin layers [14], and encapsulation in hexagonal boron nitride (hBN) keeps the plasmons confined and long-lived [15], although the phonon polaritons of hBN itself [16,17] then enter the problem. These advances make coupled-layer plasmons a tunable and optically accessible platform, but they concern the charge response of non-magnetic conductors. In a spin-split metal, the plasmon acquires a spin content, and how that content is distributed among the modes of a bilayer has not been addressed.

In altermagnetic metals, a density oscillation can be accompanied by a longitudinal spin oscillation because the Fermi surfaces are spin-split. For single-layer altermagnets, highly anisotropic spin plasmons have been predicted, and their propagation can be tuned by strain [18,19]. Collective modes of the spin channel itself, such as spin demons, require an interaction in the spin channel [20,21]. When the interaction is Coulombic and acts on the density alone, the spin signal of a charge plasmon is fixed by the density–spin mixed response of each layer, and the bilayer leaves two kinds of questions open. The first concerns the control variables. The layer index adds the relative magnetic domain of the two layers, which sets the sign with which the two layer contributions add in the total spin signal, as the interlayer phase does in other bilayer and thin-film systems [22,23,24], and domain patterning and field-assisted thermal cycling have been demonstrated in MnTe [25]. The relative domain, the propagation direction, the electron filling, and the dielectric environment are therefore candidate control variables, but it is not known which of them selects the plasmon that appears in the spin channel, which sets its energy, and which survives the phonon bands of hBN that overlap the plasmon energies in the mid-infrared. In a multi-orbital metal, moreover, the number of carriers differs from the current weight near the Fermi level, so the filling alone cannot be used to infer either the plasmon energy or its spin signal. The second is a discrimination problem. A missing spin peak is ambiguous, because it may reflect a vanishing local coupling between density and spin or only a cancelation between the layers, and no readout that separates the two has been identified.

Here, we identify the control variables and a discriminating readout for a Coulomb-coupled ${\mathrm{RbV}}_{2}$${\mathrm{Te}}_{2}$O bilayer. We describe each layer by the four-band tight-binding model with spin–orbit coupling and compute the density–longitudinal-spin response in the random-phase approximation (RPA). We find that (i) the relative domain selects which plasmon appears in the total-spin channel and leaves the charge spectra unchanged; (ii) the layer-difference spin discriminates interlayer cancelation from a node along the diagonal propagation direction; (iii) the electron filling tunes the plasmon energy and the relative spin amplitude together, through the orbital composition of the current; and (iv) these results persist with an hBN spacer, with a leakage of only about 2%. The Coulomb interaction acts in the density channel only, so the spin spectra describe the spin projection of the charge plasmons and not collective modes of the spin channel. The model is a representative description of the low-energy electronic structure of ${\mathrm{RbV}}_{2}$${\mathrm{Te}}_{2}$O: the symmetry-based results hold for any parameters that preserve the symmetry relations of Section 2.3, whereas the plasmon energies and their filling dependence belong to the chosen parameters. The scope and the approximations of the calculation are summarized in Section 3.6.

## 2. Materials and Methods

We describe each electronic layer by the four-band tight-binding model of ${\mathrm{RbV}}_{2}$${\mathrm{Te}}_{2}$O with spin–orbit coupling (SOC) and couple the two layers through the Coulomb interaction only. The density–longitudinal-spin response of the bilayer is calculated in the random-phase approximation (RPA) from the bare response of each layer, with the Coulomb interaction acting in the density channel (Section 2.2). Time reversal and the exchange of momentum components give symmetry relations that determine how the relative domain and the propagation direction enter the spin signal (Section 2.3). An orbital-resolved current stiffness connects the plasmon energy and the relative spin amplitude to the electron filling (Section 2.4), and an energy-dependent Coulomb kernel represents the hBN spacer (Section 2.5). The numerical settings are given in Section 2.6.

### 2.1. Electronic Structure and Bilayer Domain Configurations

**Figure 1:**
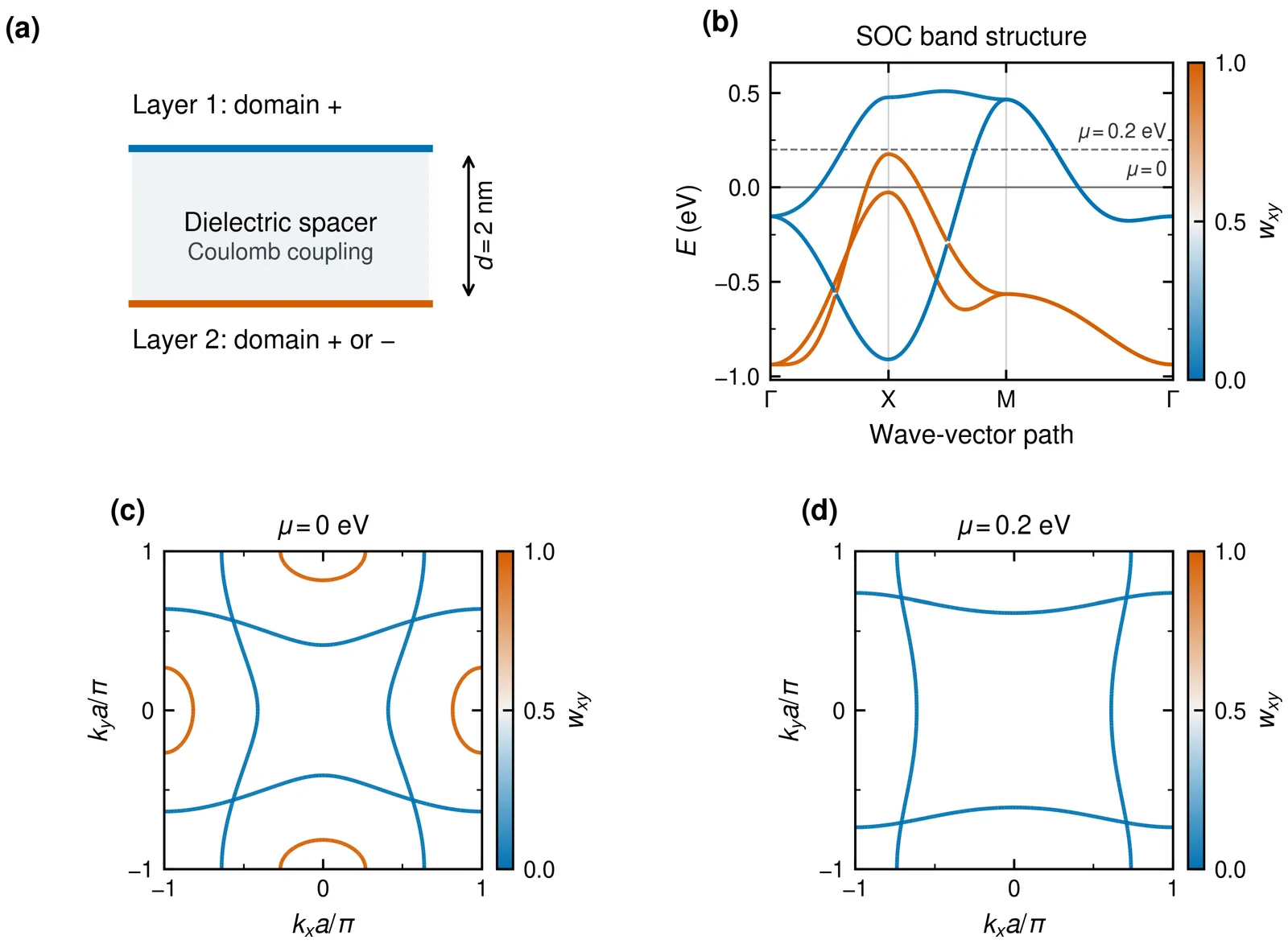
${\mathrm{RbV}}_{2}$${\mathrm{Te}}_{2}$O Coulomb bilayer and orbital-resolved electronic structure. (**a**) Two electronic layers separated by an insulating spacer with a charge-plane separation of 2 nm; both layers have the same lattice orientation. (**b**) Bands with spin–orbit coupling (SOC) along Γ–X–M–Γ, colored by *xy* weight; horizontal lines mark μ = 0 and 0.2 eV. (**c**,**d**) Fermi contours at the corresponding chemical potentials, colored by *xy* orbital weight. The contours were extracted from a 401 × 401 energy grid and colored with the eigenstates at the contour points.

Each electronic layer is described by the four-band tight-binding model of ${\mathrm{RbV}}_{2}$${\mathrm{Te}}_{2}$O with spin–orbit coupling (SOC) of Ref. [7], with in-plane lattice constant *a* = 4.020 Å. The low-energy basis consists of orbitals on the two V sublattices,

(1) $$\{d_{xz,A,↑},\;d_{xy,A,↑},\;d_{yz,B,↓},\;d_{xy,B,↓}\},$$

where *A* and *B* label the sublattices, and the arrows indicate the spin along the magnetic axis. In this basis, the spin and the sublattice are nearly interchangeable labels. The on-site energies of the *xz*/*yz* and *xy* orbitals are −0.05224 and −0.5296 eV. The nearest-neighbor hoppings of the *xz* orbital along the two axes are −0.2508 and 0.09618 eV, and those of the *xy* orbital are −0.07194 and −0.02132 eV. The two SOC parameters are 0.3862 and 1.168 meV. The remaining hoppings and the sign conventions follow Ref. [7], and we use the original fitted parameters without modification. The parameters of Ref. [7] were fitted to first-principles bands of ${\mathrm{RbV}}_{2}$${\mathrm{Te}}_{2}$O and checked against experiments on Rb-intercalated $V_{2}$${\mathrm{Te}}_{2}$O. We have not refitted them to the Rb-deficient compound, and we do not claim that the model is a validated quantitative description of ${\mathrm{Rb}}_{1-\delta }$$V_{2}$${\mathrm{Te}}_{2}$O. The chemical potential μ is measured from the energy zero of the model, and its relation to the Rb content of real samples is discussed in Section 3.6. The orbital-resolved bands along Γ–X–M–Γ are shown in Figure 1b, and the Fermi contours at two fillings are shown in Figure 1c,d, with the orbital weights obtained from the SOC eigenstates.

The single-layer density operator is *N* = $I_{4}$ and the longitudinal spin operator is *S* = 2*S*_z_/*ℏ* = diag(1, 1, −1, −1). The time-reversed domain, denoted “−” with respect to the reference domain “+”, satisfies

(2) $$H_{-}\left(k\right)=H_{+}{\left(-k\right)}^{*},\;S_{-}^{\mathrm{orb}}=-S_{+}^{\mathrm{orb}},$$

where $S_{\pm }^{\mathrm{orb}}$ is the physical spin matrix in the orbital basis of each domain, so that both domains refer to the same physical spin direction. The two layers have the same lattice orientation, and we label the domain configurations ++, +−, −+ and −−, specifying the domains of the first and second layer.

The layers are separated by an insulator, and electron tunneling between them is neglected, so that they interact only through the Coulomb interaction. Their distance is the charge-plane separation *d*. For the comparisons of domain, direction, and environment, both layers have the same chemical potential μ, measured from the energy zero of the tight-binding model. For the calculations with a one-sided source, we use $\mu_{2}$ = − $\mu_{1}$.

### 2.2. Density and Longitudinal-Spin Response in the Random-Phase Approximation

The band energies $E_{nk}$ and eigenstates $u_{nk}$ of the single-layer Hamiltonian determine the bare response. For an excitation energy *E* and in-plane wave vector **q**, with $k_{\pm }=k\pm q/2$ and vertex matrix elements $M_{nm}^{a}=u_{nk_{-}}^{†}O_{a}u_{mk_{+}}$ for $O_{a}\in \left\{N,S\right\}$, the bare response reads [8,26]

(3) $$\Pi_{ab}\left(q,E\right)=\frac{1}{A}\sum_{k,n,m}\frac{\left[f\left(E_{nk_{-}}\right)-f\left(E_{mk_{+}}\right)\right]M_{nm}^{a}\;{\left(M_{nm}^{b}\right)}^{*}}{E+i\eta +E_{nk_{-}}-E_{mk_{+}}}.$$

Here, *A* is the in-plane area, *f* is the Fermi function at the given temperature and chemical potential, and η > 0 is a numerical broadening. The sum contains all intraband and interband transitions of the four-band model, and the density vertex uses the point-orbital approximation in the atomic Bloch gauge. We write $p=\Pi_{NN}$ for the density response and $r=\Pi_{SN}$, $c=\Pi_{NS}$ for the mixed density–spin responses. The mixed responses are the only channel through which a charge excitation enters the spin signal.

The bare responses of the two layers are arranged in a block-diagonal matrix Π in the basis ($N_{1}$, $S_{1}$, $N_{2}$, $S_{2}$), and the Coulomb matrix $V_{N}$ acts in the density subspace. The RPA response is

(4) $$\chi ={\left(I-\Pi V\right)}^{-1}\Pi ,$$

where *V* equals $V_{N}$ in the density subspace and vanishes elsewhere. The Coulomb interaction therefore acts only in the density channel, and no interaction is included in the spin channel. The spin spectra are thus the spin projection of the charge plasmons, which is supplied by *r* and *c*. Collective modes of the spin channel itself, such as spin demons, are outside this model. For a probe vector *w*, the spectral function is Lw=−Im(w†χw). The layer-sum and layer-difference probes $N_{1}$ ± $N_{2}$ and $S_{1}$ ± $S_{2}$ are unnormalized, whereas the dynamic-hBN spectra use the unit-normalized probes $\left(N_{1}\pm N_{2}\right)/\sqrt{2}$ and $\left(S_{1}+S_{2}\right)/\sqrt{2}$; the two conventions differ by a factor of 2.

In a symmetric static environment with dielectric constant κ, the bilayer density interaction is

(5) $$V_{N}=\frac{2\pi \;(14.40\;\mathrm{eV}\;Å)}{\kappa \;q_{A}}\left(\begin{array}{cc} 1 & e^{-qd}\\ e^{-qd} & 1 \end{array}\right),$$

where $q_{A}$ is the wave-vector modulus in ${Å}^{-1}$, *q* and *d* are in consistent units, and $V_{N}$ is in eV ${Å}^{2}$. For equal layer responses $p_{1}=p_{2}=p$, the layer-even and layer-odd channels have the interaction eigenvalues $v_{\pm }=V_{11}\pm V_{12}$, which correspond to the optical and acoustic charge branches [10]. The representative energy of each branch is given by the real-axis condition

(6) Re1−v±(q,E)p(q,E)=0,

and its spectral intensity follows from Equation (4). The propagation angle φ is measured from the crystal *x* axis, so that φ = $45^{\circ }$ is the diagonal direction.

For the total spin $S_{+}=S_{1}+S_{2}$, the Coulomb correction takes a layer-parity form (derived in Section S1 of the Supplementary Materials),

(7) $$\Delta \chi_{S_{+}S_{+}}=\sum_{\alpha =\pm }\frac{\left(r_{1}+\alpha r_{2}\right)\left(c_{1}+\alpha c_{2}\right)}{2}\;\frac{v_{\alpha }}{1-p\;v_{\alpha }}.$$

Equation (7) organizes the results of Section 3. The denominator $1-p\;v_{\alpha }$ fixes the charge branch and its position, while the numerator fixes how much of that branch is visible in the total-spin channel. We call a branch bright in a given readout channel if it has a spectral peak there, and dark if it has none.

### 2.3. Symmetry Relations for Domain and Propagation Direction

Two symmetry relations constrain the numerator of Equation (7). The first follows from time reversal, which preserves *N* and reverses *S*. The responses of the two time-reversed domains satisfy

(8) $$\Pi_{ab}^{-}\left(q,E\right)=\epsilon_{a}\epsilon_{b}\;\Pi_{ba}^{+}(-q,E),\;\epsilon_{N}=1,\;\epsilon_{S}=-1.$$

With the inversion symmetry of the model, Equation (8) leaves *p* unchanged and reverses the sign of *r* and *c* in the opposite domain. In the same domain we thus have $r_{1}$ = $r_{2}$ and $c_{1}$ = $c_{2}$, and in opposite domains $r_{2}$ = −$r_{1}$ and $c_{2}$ = −$c_{1}$.

The second relation follows from the exchange of the momentum components. In the four-orbital basis the unitary matrix $U=\mathrm{diag}\left(i,i,1,1\right)\;P_{\mathrm{orb}}$, where $P_{\mathrm{orb}}$ is the orbital permutation that exchanges *x* and *y*, satisfies $H\left(k_{x},k_{y}\right)=UH\left(k_{y},k_{x}\right)U^{†}$, $UNU^{†}=N$ and $USU^{†}=-S$. Exchanging $k_{x}↔k_{y}$ in the integral of Equation (3) gives

(9) $$\Pi_{SN}\left(q_{x},q_{y},E\right)=-\Pi_{SN}(q_{y},q_{x},E),$$

and the same relation holds for $\Pi_{NS}$ (Section S2). For $q_{x}=q_{y}$, that is, for propagation along the diagonal (φ = $45^{\circ }$), *r* and *c* vanish, while *p* does not. We also verify numerically in Section S2 that the full bare response matrices of the opposite domain equal those of the reference domain rotated by $90^{\circ }$ to within 3 × $10^{-15}$. This holds because of the crystal symmetry and an isotropic in-plane Coulomb environment.

### 2.4. Orbital-Resolved Current Stiffness and Relative Spin Weight

At long wavelength, the position of a charge branch is set by the low-energy current response. To connect it to the filling, we compute the orbital-resolved current stiffness. With *x* = $k_{x}$*a*, the band slope is $u_{nk,x}=\partial E_{nk}/\partial x$ (in eV) and the group velocity is $v_{nk,x}=a\;u_{nk,x}/\hbar$. The orbital weights follow from the eigenstate coefficients, $w_{nk}^{xy}={\left|c_{xy,A}\right|}^{2}+{\left|c_{xy,B}\right|}^{2}$ and $w_{nk}^{xz/yz}=1-w_{nk}^{xy}$. On an $N_{k}\times N_{k}$ grid, the orbital density of states and the velocity-weighted energy kernel are

(10) $$g_{\alpha }\left(\varepsilon \right)=\frac{1}{N_{k}^{2}}\sum_{n,k}w_{nk}^{\alpha }\;\delta (\varepsilon -E_{nk}),$$

(11) $$K_{\alpha }\left(\varepsilon \right)=\frac{1}{N_{k}^{2}}\sum_{n,k}w_{nk}^{\alpha }\;u_{nk,x}^{2}\;\delta (\varepsilon -E_{nk}),$$

with α ∈ {*xy*, *xz*/*yz*}, in units of ${\mathrm{eV}}^{-1}$ and eV, respectively. The orbital current stiffness at finite temperature is

(12) $$D_{xx}^{\alpha }\left(\mu ,T\right)=\int d\varepsilon \;K_{\alpha }\left(\varepsilon \right)\left[-\frac{\partial f(\varepsilon -\mu )}{\partial \varepsilon }\right],$$

and the total stiffness $D_{xx}=D_{xx}^{xy}+D_{xx}^{xz/yz}$ can also be obtained directly from a momentum sum,

(13) $$D_{xx}=\frac{1}{N_{k}^{2}}\sum_{n,k}\frac{f_{nk}\left(1-f_{nk}\right)}{k_{B}T}\;u_{nk,x}^{2}.$$

The orbital decomposition uses the weights of the SOC-mixed eigenstates, and the components add up to the total. To separate changes in the state weight from changes in the velocity during a filling scan, we define the thermal-window state weight

(14) $$G_{T}=\int d\varepsilon \;g\left(\varepsilon \right)\left(-\frac{\partial f}{\partial \varepsilon }\right),\;D_{xx}=G_{T}\;{\langle u_{x}^{2}\rangle }_{T},$$

with $g=g_{xy}+g_{xz/yz}$. The leading long-wavelength intraband term of the density response along *x* gives

(15) $$\Pi_{NN}≃\frac{D_{xx}\;q_{A}^{2}}{E^{2}},\;E_{\pm }^{\mathrm{LW}}=q_{A}\sqrt{v_{\pm }D_{xx}},$$

so that, for a fixed dielectric environment and geometry, the acoustic energy scales as $\sqrt{D_{xx}}$.

The size of the numerator is connected to the current stiffness in the same way. With the eigenstate spin expectation value $s_{nk}=u_{nk}^{†}Su_{nk}$, we define the spin-weighted stiffness

(16) $$D_{ij}^{s}=\frac{1}{N_{k}^{2}}\sum_{n,k}\left[-\frac{\partial f}{\partial E_{nk}}\right]s_{nk}\;u_{nk,i}\;u_{nk,j}.$$

In the leading intraband long-wavelength limit, the density-driven relative spin amplitude along the propagation direction φ is

(17) $$\frac{\Pi_{SN}}{\Pi_{NN}}≃\frac{D_{\phi \phi }^{s}}{D_{\phi \phi }},\;P\equiv \frac{D_{xx}^{s}}{D_{xx}}\;\;(\phi =0^{\circ }).$$

SOC enters through the eigenstate spin expectation values. Equations (15) and (17) are analytic estimates of the trends; the finite-wave-vector results are obtained from Equations (3) and (4). In Section 3.3, we compare $P^{2}$ with $|\Pi_{SN}/\Pi_{NN}{|}^{2}$ of the full response at the acoustic real-axis crossing. The long-wavelength expansion is given in Section S2.

### 2.5. hBN Dielectric Environment and Asymmetry Measure

In the dynamic calculations, the insulator between the two layers is hBN. Its in-plane and out-of-plane phonon responses differ, and the resulting anisotropic dielectric tensor modifies the interlayer field and the Coulomb coupling [16,27]. In a local quasi-static approximation, we construct the energy-dependent kernel $V_{N}\left(q,E\right)$ from the Green’s function of the layered medium (Section S6). The two principal components of the dielectric tensor have the Lorentz form

(18) $$\epsilon_{\alpha }\left(E\right)=\epsilon_{\infty ,\alpha }\left[1+\frac{{\tilde{\nu }}_{\mathrm{LO},\alpha }^{2}-{\tilde{\nu }}_{\mathrm{TO},\alpha }^{2}}{{\tilde{\nu }}_{\mathrm{TO},\alpha }^{2}-{\left(E/hc\right)}^{2}-i\gamma_{\alpha }E/hc}\right],$$

where α∈ {‖, ⊥} denotes the in-plane and out-of-plane components, ${\tilde{\nu }}_{\mathrm{TO}}$ and ${\tilde{\nu }}_{\mathrm{LO}}$ are the wavenumbers of the transverse and longitudinal optical phonons, γ is the damping, and *hc* = 1.240 × $10^{-4}$ eV cm. We use the parameters of Ref. [27]: in plane, ε*∞* = 4.87, ${\tilde{\nu }}_{\mathrm{TO}}/{\tilde{\nu }}_{\mathrm{LO}}$ = 1370/1610 ${\mathrm{cm}}^{-1}$ and γ = 5 ${\mathrm{cm}}^{-1}$; out of plane, ε*∞* = 2.95, 780/830 ${\mathrm{cm}}^{-1}$ and γ = 4 ${\mathrm{cm}}^{-1}$. The two phonon bands span approximately 96.7–102.9 meV and 169.9–199.6 meV. The outer media are lossless scalar dielectrics, and the interfacial gaps have a dielectric constant of 1.

All structures have a charge-plane separation of 2 nm. The gapless structure has 2 nm of hBN and outer dielectric constants of 4. The symmetric gapped structure has 0.3 nm gaps above and below, 1.4 nm of hBN, and outer dielectric constants of 4. The asymmetric structure has gaps of 0.1 and 0.4 nm above and below, 1.5 nm of hBN, and outer constants of 1 and 4. The static κ = 4 environment is kept as a separate reference, which isolates the electronic and interlayer-interference effects.

To quantify the dark-branch projection induced by top–bottom asymmetry, we scan the path

(19) $$\kappa_{t,b}=2.5\mp 1.5\lambda ,\;h_{t,b}=\left(0.25\mp 0.15\lambda \right)\;\mathrm{nm},$$

where $h_{t,b}$ are the top and bottom interfacial gaps and the hBN thickness is fixed at 1.5 nm. The point λ = 0 is the symmetric structure with 0.25 nm gaps and outer constants of 2.5, and λ = 1 is the asymmetric structure described above. At each real-axis energy we diagonalize the complex symmetric Coulomb kernel, $V_{N}u_{j}=v_{j}u_{j}$ with $u_{j}^{T}u_{j}=1$, and define the layer-odd-type channel *o* as the one that maximizes ${\left|e_{-}^{T}u_{j}\right|}^{2}/({\left|e_{-}^{T}u_{j}\right|}^{2}+{\left|e_{+}^{T}u_{j}\right|}^{2})$, where $e_{\pm }={\left(1,\pm 1\right)}^{T}/\sqrt{2}$. At the symmetric point, this channel reduces to the acoustic layer-odd branch. Its electronic correction to the total spin is $\Delta \chi_{o}=\left(r\;u_{o}\right)\left(u_{o}^{T}c\right)\;v_{o}/\left(1-p\;v_{o}\right)$ with $r=(\Pi_{SN}^{\left(1\right)},\Pi_{SN}^{\left(2\right)})$ and $c={\left(\Pi_{NS}^{\left(1\right)},\Pi_{NS}^{\left(2\right)}\right)}^{T}$. With L++,+−o=−ImΔχo++,+− for the same and opposite domains, the window-area ratio is

(20) $$R\left(\lambda \right)=\frac{\int_{195\;\mathrm{meV}}^{320\;\mathrm{meV}}L_{++}^{o}\left(E,\lambda \right)\;dE}{\int_{195\;\mathrm{meV}}^{320\;\mathrm{meV}}L_{+-}^{o}\left(E,\lambda \right)\;dE}.$$

It measures the residual projection of the dark branch (the same-domain layer-odd channel) relative to the bright branch (the opposite-domain layer-odd channel).

### 2.6. Numerical Settings

All calculations use the fixed-order Hamiltonian and a 300 K Fermi distribution. Unless stated otherwise, the static comparisons of domain and direction use *d* = 2 nm, κ = 4, μ = 0, *q* = 0.2 ${\mathrm{nm}}^{-1}$, φ = $0^{\circ }$ and η = 0.5 meV, with a 384 × 384 momentum grid and a 32,768-bin transfer-energy histogram. Orbital occupations and stiffnesses use a 512 × 512 grid. The filling-dependent RPA energies use a 256 × 256 grid, *q* = 0.05 ${\mathrm{nm}}^{-1}$ and η = 1 meV. The settings of all calculations are listed in Table S2. The calculations were implemented in Python 3.13 with NumPy 2.5 and SciPy 1.18, and the figures were prepared with Matplotlib 3.11. The calculation and plotting programs and the numerical data of all figures are deposited in Zenodo (Data Availability Statement). Convergence with respect to the momentum grid and the number of transfer-energy bins was tested for the plasmon energies, the relative spin amplitude, and the leakage ratio *R*: for the settings used here, the deviations of the main settings from a 1024 × 1024 grid with 131,072 bins are below 2 × $10^{-4}$%, and all grids and bin numbers tested agree with this reference within 0.002% (Section S8).

## 3. Results and Discussion

All results below are obtained for the model of Section 2 at *T* = 300 K. Unless stated otherwise, we use the static reference environment (*d* = 2 nm, κ = 4), μ = 0, *q* = 0.2 ${\mathrm{nm}}^{-1}$, propagation along *x* and η = 0.5 meV. The broadening η is a numerical parameter and is not tied to a lifetime; its influence on the bright–dark assignment is examined in Section 3.4.

### 3.1. The Relative Domain Exchanges the Spin Weight of the Acoustic and Optical Plasmons

**Figure 2:**
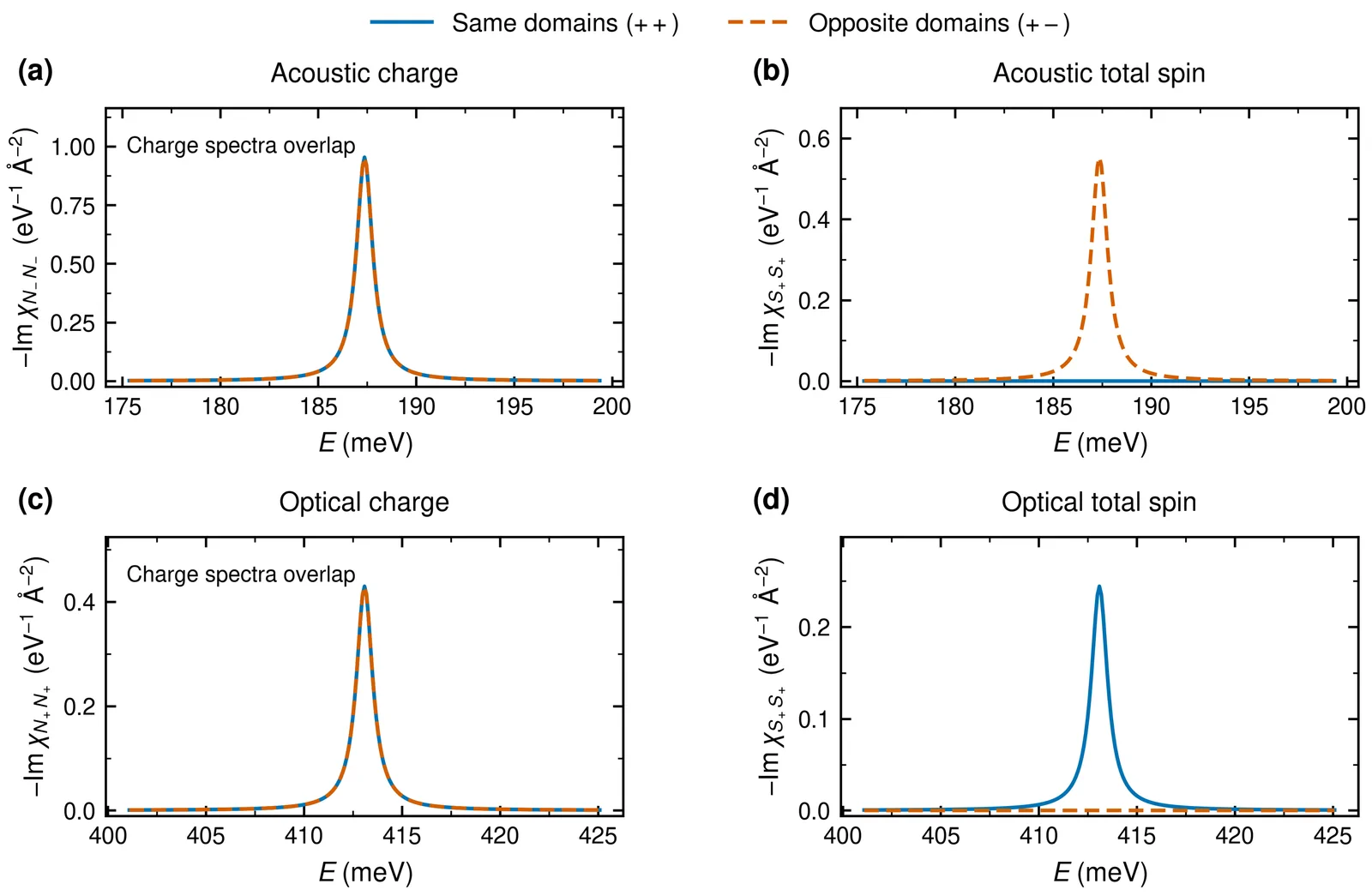
Relative domain selects the total-spin projection of the acoustic and optical plasmons. (**a**,**b**) Layer-odd charge and total-spin spectra in the acoustic window; (**c**,**d**) layer-even charge and total-spin spectra in the optical window. Blue solid lines: same domain (++); orange dashed lines: opposite domain (+−). The charge curves in (**a**,**c**) coincide for both configurations. φ = $0^{\circ }$, *q* = 0.2 ${\mathrm{nm}}^{-1}$, *d* = 2 nm, κ = 4, μ = 0, *T* = 300 K, η = 0.5 meV, 384 × 384 momentum grid. Layer-sum and layer-difference probes are unnormalized.

Figure 2 shows the layer-odd (acoustic) and layer-even (optical) charge spectra together with the total-spin spectra of the same-domain (++) and opposite-domain (+−) bilayers at equal filling in a symmetric environment. The charge spectra in Figure 2a,c coincide for the two configurations, with real-axis crossings at 187.4 and 413.1 meV. The total-spin spectra in Figure 2b,d differ strongly: the acoustic peak appears only for the opposite domain and the optical peak only for the same domain. A missing total-spin peak therefore does not signal the loss of the plasmon. The charge resonance stays at the same energy, and only its projection onto the spin is suppressed. This behavior follows from Equation (7). The denominator $1-pv_{\alpha }$ does not depend on the domain, so the charge spectra are unchanged. The numerator does. For the same domain, $r_{1}$ = $r_{2}$ and $c_{1}$ = $c_{2}$, the layer-odd term (α = −) vanishes and only the layer-even optical term survives. For the opposite domain, Equation (8) reverses the sign of the second layer; the layer-even term vanishes, and only the acoustic term is left. The relative domain thus redistributes the spin projection among plasmons that do not move.

**Figure 3:**
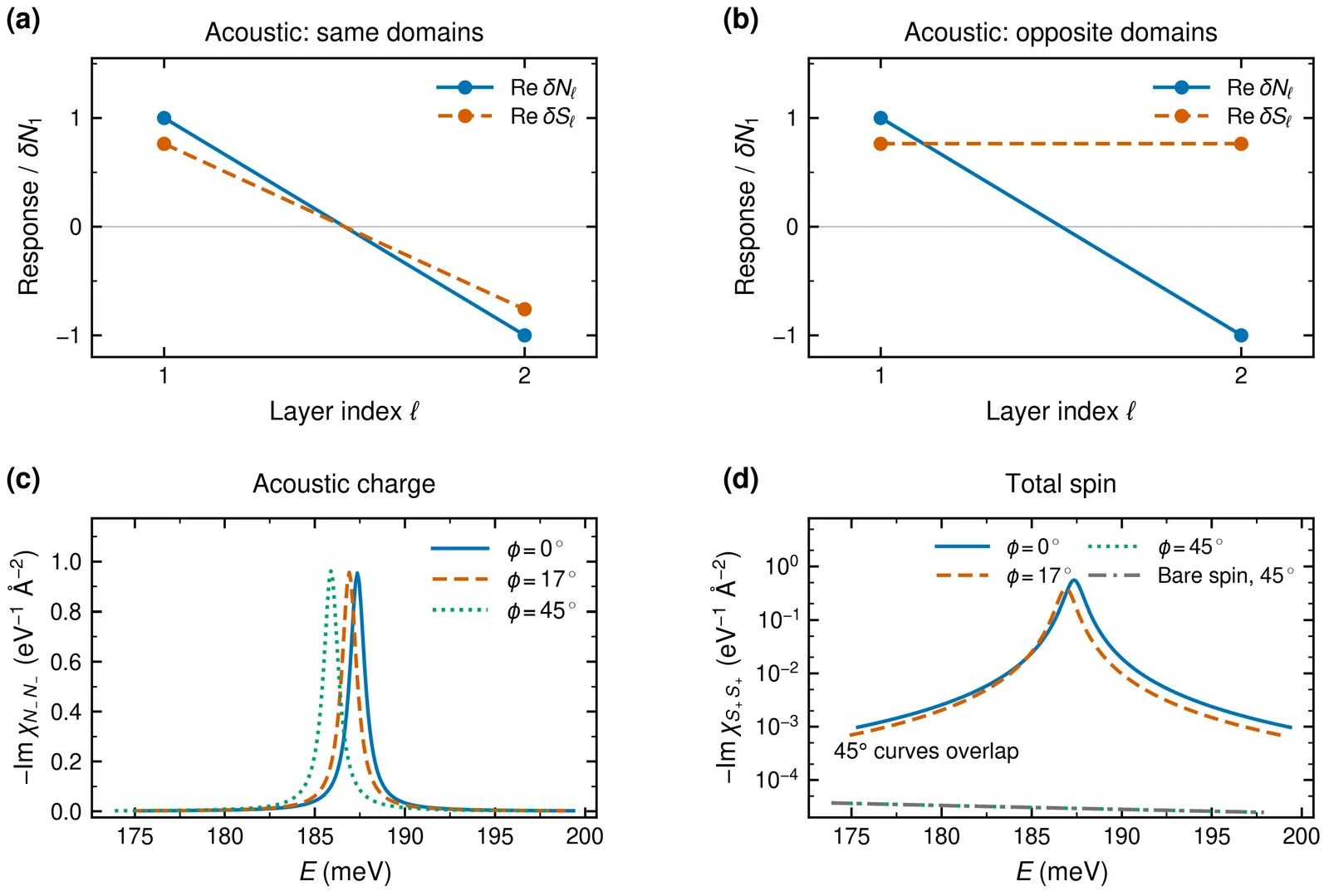
Two mechanisms of spin extinction: interlayer cancelation and a directional node. (**a**,**b**) Real parts of the layer amplitudes at the acoustic real-axis condition for the same and opposite domains, with the first-layer density amplitude normalized to 1; lines are guides to the eye. (**c**,**d**) Layer-odd charge and total-spin spectra of the opposite domain at φ = $0^{\circ }$, $17^{\circ }$ and $45^{\circ }$. Panel (**d**) has a logarithmic vertical axis, and the $45^{\circ }$ curve (green) coincides with the gray bare-spin background. Other parameters as in Figure 2.

The layer-resolved amplitudes in Figure 3a,b show the same interference. For an acoustic density drive normalized to unit amplitude in the first layer, the charge amplitudes of the two layers are (1, −1), and the real parts of the spin amplitudes are approximately (0.762, −0.762) in the same domain and (0.762, 0.762) in the opposite domain. Each layer carries a finite local spin signal in both cases, and the domain decides whether the two signals cancel or add in the total spin. The optical drive gives the complementary result (Figure S1a,b). Reading out the layer-difference spin $S_{-}=S_{1}-S_{2}$ exchanges addition and cancelation, so that the same-domain acoustic plasmon and the opposite-domain optical plasmon show a peak that fills in the dark branches of Figure 2 (Figure S1c,d). Table 1 summarizes the resulting selection rule. In this model, the opposite-domain response of a single layer is also equal to that of the reference domain rotated by $90^{\circ }$ (Section S2), so that the same rule applies to two layers with orthogonal orientations in an isotropic in-plane Coulomb environment.

*Position relative to the particle–hole continuum.* We located the continuum from the bare density response at 300 K (Section S9). The intraband particle–hole continuum of the model ends at 58.9 meV for *q* = 0.2 ${\mathrm{nm}}^{-1}$ and at 14.7 meV for *q* = 0.05 ${\mathrm{nm}}^{-1}$. All plasmons discussed in this work, namely the static peaks at 187.4 and 413.1 meV (*q* = 0.2 ${\mathrm{nm}}^{-1}$), the dynamic-hBN peaks at 214.0 and 409.3 meV, and the filling-scan peaks at *q* = 0.05 ${\mathrm{nm}}^{-1}$ (50.2 and 219.8 meV at μ = 0, 42.6 and 185.3 meV at μ = 0.2 eV), lie above this edge by a factor of 2.9 or more. Interband transitions set in at about 2 meV on the finite grid, so the plasmons lie within the energy range of interband transitions, but the bare interband spectral weight at the peak energies is very small. The widths of the peaks therefore follow the numerical broadening: the full width at half maximum equals 2η to within 0.1% for all modes except the optical mode at *q* = 0.2 ${\mathrm{nm}}^{-1}$ (excess of 0.8% at η = 0.5 meV), and a linear extrapolation to η → 0 leaves widths below $10^{-4}$ meV for the acoustic modes (Table S8). The model thus contains no appreciable intrinsic damping of these plasmons by particle–hole excitations, and the broadening η = 0.5 meV used in the calculations stands for damping by disorder, phonons, and other processes that are not computed (Section 3.6). Because the grid is discrete, this result describes the discretized response and is not a proof of the absence of damping in the continuum limit.

### 3.2. The Layer-Difference Spin Separates a Directional Node from Interlayer Cancelation

The selection rule of Section 3.1 requires finite single-layer mixed responses *r* and *c*. These vanish along the diagonal of the Brillouin zone, which gives a second origin of a missing spin peak. Figure 3c,d shows the layer-odd charge and total-spin spectra of the opposite-domain bilayer for propagation directions of $0^{\circ }$, $17^{\circ }$ and $45^{\circ }$. The acoustic crossing moves only from 187.4 to 186.9 and 185.9 meV, a change below 1%, and all three charge spectra keep a clear resonance. The total-spin resonance, in contrast, weakens with increasing angle and coincides with the bare-spin background at $45^{\circ }$. The density-driven relative spin amplitude Π_SN_/Π_NN_ is 0.762, 0.634 and 0 at $0^{\circ }$, $17^{\circ }$ and $45^{\circ }$, following approximately cos 2φ (Figure S2). The logarithmic scale of Figure 3d shows that the collective enhancement of the charge branch is gone at $45^{\circ }$, while a finite bare spin background remains.

The node is exact and follows from the symmetry of the model. For the SOC four-band Hamiltonian, exchanging $k_{x}$ and $k_{y}$ together with the basis transformation *U* preserves the Hamiltonian and the density vertex and reverses the spin vertex, so that Π_SN_ and Π_NS_ vanish at $q_{x}$ = $k_{y}$ (Equation (9)), while the density response remains finite. The cancelation involves symmetry-related transitions over the whole Brillouin zone and does not depend on any phase between the layers. The finite background at $45^{\circ }$ is the bare response. At 186 meV, the bare spin and density losses differ by about 0.22%, the interband part is only 0.23% of the bare spin loss, and the intraband spin background persists and equals the density background when SOC is switched off (Section S5). The node therefore removes the collective projection of the Coulomb branch and leaves the intraband tail of the broadened bare response, and SOC does not break it. Anisotropic spin plasmons and their directional tuning have been reported for single-layer altermagnets [18,19]. In the bilayer, the node gives a second kind of dark total-spin peak, which is distinguished from interlayer cancelation by the layer-difference spin. In the same-domain acoustic branch, the local spin amplitudes of the two layers are finite and opposite, so the total spin is dark, and the layer-difference spin is bright. Along $45^{\circ }$, the mixed responses of both layers vanish, and both spin channels lose the collective peak. The relative domain thus acts on how the two layers combine, whereas the propagation direction acts on the local coupling itself.

*Relative twist of the layers.* The two layers are assumed to be aligned in the calculations above. We tested a rotation of the second layer by θ about the *z* axis, keeping the Coulomb coupling and the spin axis fixed, so that the response of the second layer is evaluated at the wave vector rotated into its crystal frame (Section S13). For θ = 0, the results above are reproduced, and for θ = $90^{\circ }$, the same-domain result equals the opposite-domain result at θ = 0 to within $10^{-13}$. For propagation along *x* (φ = $0^{\circ }$), the ratio of the total-spin area of the dark branch to that of the bright branch is 0.04–0.05% at θ = 0 and remains below 0.7% for θ ≤ $5^{\circ }$ at φ = $0^{\circ }$ and 22.$5^{\circ }$. It is 0.13–0.15% at $10^{\circ }$, 0.5–0.6% at $15^{\circ }$, about 3% at 22.$5^{\circ }$ and 11% at $30^{\circ }$, and reaches 100% at $45^{\circ }$, where the two domain configurations are no longer distinguished; between $60^{\circ }$ and $90^{\circ }$ the roles of the two branches are exchanged. The growth follows the projection weights ${\left[\cos2\varphi \mp \cos2\left(\varphi -\theta \right)\right]}^{2}$, whose ratio for φ = $0^{\circ }$ is $\tan^{4}$θ. The twist also shifts the peaks by up to 0.7 meV for the acoustic and 0.3 meV for the optical plasmon at θ = $45^{\circ }$. A twist of a few degrees therefore does not change the selection, whereas a larger twist redistributes the spin weight between the two plasmons.

### 3.3. Electron Filling Softens the Acoustic Plasmon and Raises the Relative Spin Amplitude

**Figure 4:**
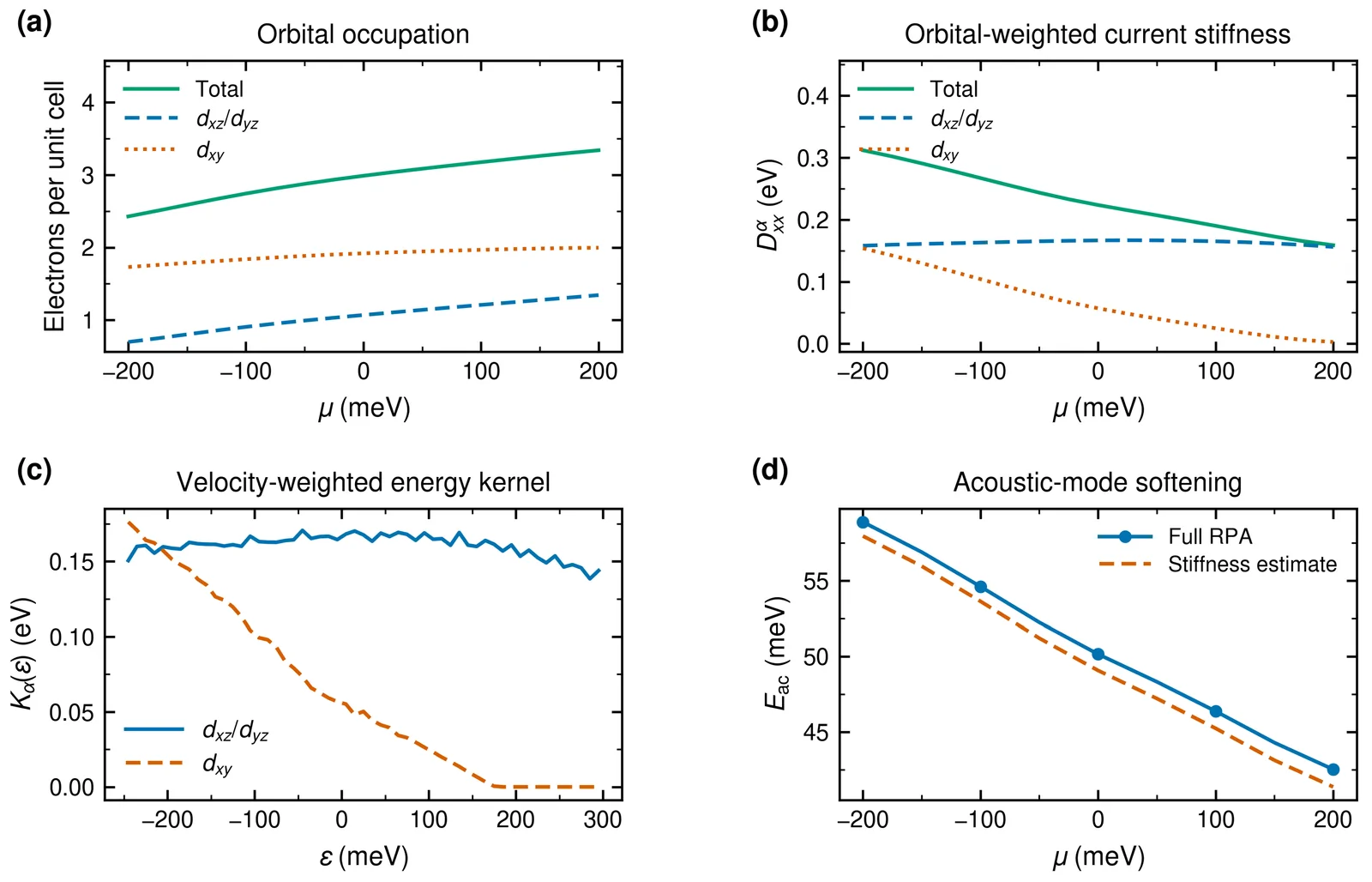
Multi-orbital filling and acoustic softening. (**a**) Total electron occupation per unit cell and orbital-weight components; (**b**) total current stiffness and its orbital decomposition; (**c**) velocity-weighted energy kernel $K_{\alpha }$ of Equation (11), with the 2 meV raw bins merged into 10 meV bins for display; (**d**) acoustic real-axis crossing of the random-phase approximation (RPA) response compared with the long-wavelength estimate of Equation (15). Occupation and stiffness use a 512 × 512 grid; the RPA energies use a 256 × 256 grid, *q* = 0.05 ${\mathrm{nm}}^{-1}$ and η = 1 meV. *T* = 300 K, *d* = 2 nm, κ = 4. The wave vector of Figure 2 is 0.2 ${\mathrm{nm}}^{-1}$.

The selections of Section 3.1 and Section 3.2 act on the numerator of Equation (7). The plasmon energies are set by the denominator and, through the current response, by the electron filling. Figure 4a,b shows the occupation per unit cell and the current stiffness $D_{xx}$ as functions of the chemical potential, together with their orbital components. The occupation increases, while $D_{xx}$ decreases: from μ = 0 to 0.2 eV, the occupation grows from 2.989 to 3.340, whereas $D_{xx}$ drops from 0.2237 to 0.1589 eV, a decrease of about 29%. Adding electrons therefore does not harden the plasmon in this model. In a multi-orbital lattice, the number of carriers differs from the low-energy current response, because the stiffness is determined by the velocity-weighted states in the thermal window, and electrons added in deeply occupied states can come with a loss of low-energy weight.

The orbital decomposition in Figure 4a,b locates the decrease in the *xy* orbital. Over the same interval, the *xy* occupation increases from 1.919 to 1.997, close to its capacity of 2, and the *xy* stiffness drops from 0.0571 to 0.00278 eV (−95.1%), whereas the *xz*/*yz* stiffness changes only from 0.1666 to 0.1562 eV. The decrease in the *xy* stiffness accounts for 83.9% of the total decrease, so the added electrons mainly enter the *xz*/*yz* component, while the loss of low-energy *xy* current weight drives the softening. The energy kernels in Figure 4c show why: the velocity-weighted *xy* kernel decays strongly with energy and approaches zero near 0.2 eV, whereas the *xz*/*yz* kernel remains finite in the same range. According to Equation (12), raising μ moves the thermal Fermi window towards energies at which the *xy* kernel is small. The Fermi contours in Figure 1c,d show the corresponding geometry: the *xy*-dominated contours present at μ = 0 are absent at μ = 0.2 eV, where the remaining contours carry mainly *xz*/*yz* weight. A small residual *xy* stiffness remains from SOC mixing and thermal convolution. The decomposition of Equation (14) gives a decrease of 27.5% in *G*_T_ and only 2.0% in 〈$u_{x}^{2}$〉_T_ between μ = 0 and 0.2 eV, which together account for the decrease of about 29% in $D_{xx}$. The softening is thus a loss of active state weight in the thermal window and not a decrease in the mean-squared velocity.

Figure 4d compares the acoustic real-axis crossing of the full RPA response with the long-wavelength estimate of Equation (15), $E_{\mathrm{ac}}^{\mathrm{LW}}\propto \sqrt{D_{xx}}$, which predicts a decrease of 15.7% for the stiffness change above. The full response at *q* = 0.05 ${\mathrm{nm}}^{-1}$ falls from 50.15 to 42.52 meV (−15.2%), in agreement with the stiffness picture.

**Figure 5:**
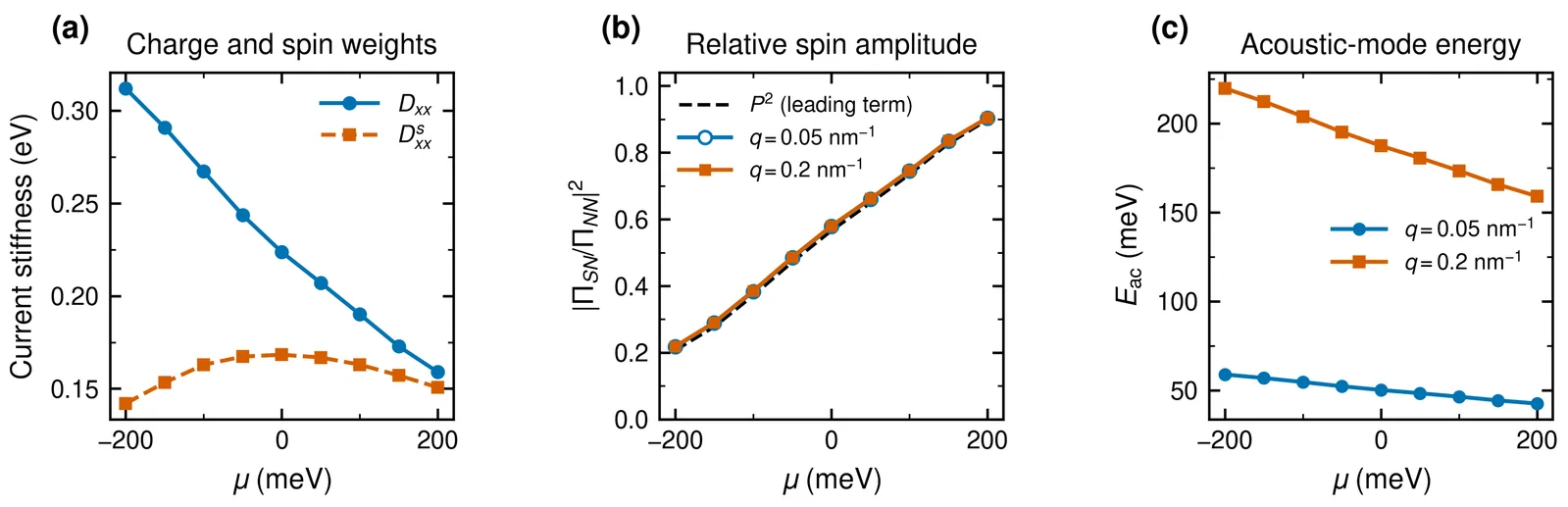
Filling tunes current stiffness, relative spin amplitude, and acoustic energy. (**a**) Charge and spin-weighted stiffness; (**b**) long-wavelength $P^{2}$ and $|\Pi_{SN}/\Pi_{NN}{|}^{2}$ of the full response at the acoustic real-axis crossing; (**c**) acoustic energy at two wave vectors. φ = $0^{\circ }$, *d* = 2 nm, κ = 4, *T* = 300 K. (**a**) 512 × 512 grid; (**b**,**c**) 256 × 256 grid and η = 0.5 meV. Figure 4d uses η = 1 meV.

Filling also changes the spin signal carried by the plasmon. Figure 5a shows that the spin-weighted stiffness $D_{xx}^{s}$ decreases only from 0.1683 to 0.1507 eV between μ = 0 and 0.2 eV, whereas $D_{xx}$ decreases much more, so that *P* = $D_{xx}^{s}$/$D_{xx}$ rises from 0.7524 to 0.9479 and $P^{2}$ from 0.566 to 0.899. By orbital, the ratio of spin-weighted to total stiffness at μ = 0 is 0.367 for *xy* and 0.884 for *xz*/*yz*, and the *xz*/*yz* ratio increases to 0.959 at 0.2 eV. The removal of the low-energy *xy* current, which has the smaller ratio, and the growth of the *xz*/*yz* ratio both raise the relative spin weight of the total current. Figure 5b,c shows that the full response at *q* = 0.2 ${\mathrm{nm}}^{-1}$ follows the same trend: while the acoustic energy falls from 187.4 to 159.0 meV, |Π_SN_/Π_NN_${|}^{2}$ rises from 0.580 to 0.904. This ratio is the squared spin amplitude per unit density amplitude, so it is normalized by the charge response, and its increase does not by itself mean a larger absolute spin signal. We therefore also computed the integrated spectral weight W=(1/π)∫(−Imχ)dE of the spin and charge peaks of the opposite-domain acoustic and the same-domain optical plasmon, over a window of ±5 full widths at half maximum around each peak (Section S15). Between μ = 0 and 0.2 eV, the spin weight increases by 30.5–33.6% at *q* = 0.05 and 0.2 ${\mathrm{nm}}^{-1}$, whereas the charge weight decreases by 15.7–16.3%. The ratio of the two weights rises from 0.58 to 0.90 for the acoustic and from 0.57 to 0.90 for the optical plasmon, in agreement with the squared vertex ratio. Both effects therefore contribute to the larger relative amplitude: the charge response of the plasmon falls, and its spin response rises. The integrated weight at finite *q* is not set by the spin-weighted stiffness alone, which decreases between μ = 0 and 0.2 eV. These weights are areas of the model spectra at η = 0.5 meV without background subtraction and are not calibrated experimental counts. At μ = 0, *P* = 0.7524 and the finite-*q* amplitude of 0.7616 (the 0.762 of Figure 3a,b) differ by about 1%, which shows that the full intraband response contains information beyond the leading Drude term. The angular dependence and the long-wavelength form $P^{2}$$\cos^{2}$(2φ) are compared in Figure S2, and Tables S3 and S4 list the values at all fillings and the orbital decomposition of the stiffness.

Comparing the full response with one that keeps only intraband transitions, the charge-window areas differ by less than 0.03% at all tested fillings and wave vectors (Table S1), so the stiffness picture applies. The same orbital current weights therefore control both quantities: the total stiffness $D_{xx}$ sets the plasmon energy, and the ratio *P* sets the relative spin amplitude. Because the *xy* current weight is lost faster than the spin-weighted part, a higher filling lowers the energy and raises the relative spin amplitude at the same time. The scan from 0 to 0.2 eV corresponds to an increase of about 2.18 × $10^{14}$${\mathrm{cm}}^{-2}$ in the areal electron density.

*Relation to the Lifshitz transition.* The Fermi surface of the model changes topology inside the scanned interval. The *xy*-type pockets at the X and Y points have their band top at μL = 175.2 meV and disappear there, and the number of periodic Fermi contours decreases from three to two between 175 and 180 meV (Section S12). A second *xy*-type pocket had already disappeared at −27.3 meV and is absent at μ = 0. The softening does not start at this transition. The derivative of the long-wavelength plasmon energy with respect to μ is already −48 meV/eV at μ = −0.1 eV and has nearly the same value at 300, 100 and 30 K (−48.4, −49.3 and −49.1 meV/eV), whereas the transition at 175.2 meV produces only a local feature in the curvature near μ = 175 meV, which the thermal broadening (*k*BT = 25.9 meV at 300 K) smooths. The transition therefore removes the *xy* current weight that remains at high filling, and the decrease in the stiffness is a continuous consequence of the energy dependence of the velocity-weighted kernel (Figure 4c).

*Moderate carrier-density changes.* A change of 2.18 × $10^{14}$ ${\mathrm{cm}}^{-2}$ is large, and we therefore also computed the response for smaller changes at fixed Hamiltonian (Section S11). For electron densities increased by 1, 3, 5 and 10 × $10^{13}$${\mathrm{cm}}^{-2}$ (μ = 7.9, 24.3, 41.3 and 85.4 meV) the acoustic RPA energy at *q* = 0.05 ${\mathrm{nm}}^{-1}$ decreases by 0.59%, 1.8%, 3.0% and 6.4%, the stiffness $D_{xx}$ by 1.2%, 3.7%, 6.1% and 12.7%, and the relative spin amplitude |Π_SN_/Π_NN_${|}^{2}$ increases by 2.3%, 7.0%, 11.8% and 24.4%. A decrease in density by the same amounts changes the energy by +0.59% to +6.0% and lowers the relative spin amplitude by 2.3% to 23%. The trend of this section therefore sets in at small density changes, and its size scales approximately with the density change in this range.

### 3.4. Dynamic hBN Shifts the Plasmon Energies and Preserves the Domain Selection

**Figure 6:**
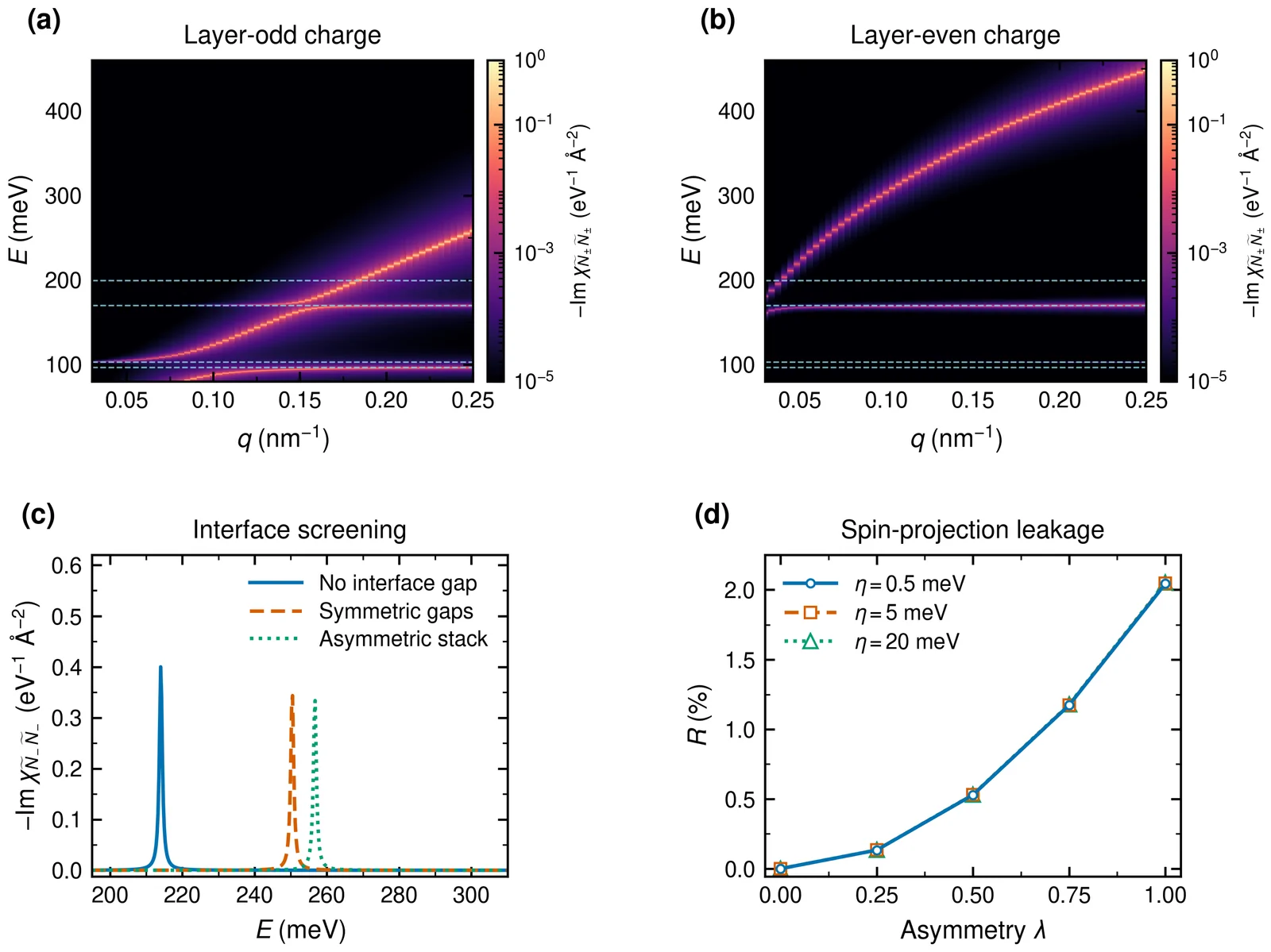
Dynamic hexagonal boron nitride (hBN) charge spectra, interfacial screening and asymmetric spin leakage. (**a**,**b**) Unit-normalized layer-odd and layer-even charge spectra of the gapless symmetric structure; the color scale is the base-10 logarithm of the loss (response units ${eV}^{-1}$${Å}^{-2}$), and dashed lines mark the two transverse/longitudinal optical (TO/LO) phonon boundaries. (**c**) Layer-odd charge spectra of the three structures, with dimensions given in Section 2.5. (**d**) Layer-odd branch window-area ratio of Equation (20); curves for different η nearly coincide. The scan path is Equation (19), and λ = 1 corresponds to the green curve in (**c**). μ = 0, φ = $0^{\circ }$, *T* = 300 K, *d* = 2 nm; (**a**,**b**) 192 × 192 grid, η = 0.5 meV, 61 *q* points; (**c**) 512 × 512 grid, *q* = 0.2 ${nm}^{-1}$, η = 0.5 meV; (**d**) 384 × 384 grid, *q* = 0.2 ${\mathrm{nm}}^{-1}$.

The phonon response of hBN makes the Coulomb kernel energy-dependent and changes the denominator of Equation (7). Figure 6a,b shows the layer-odd and layer-even charge spectra of the gapless symmetric structure as functions of energy and wave vector. Besides the main plasmon peaks that disperse with *q*, near-horizontal structures and a redistribution of spectral weight appear around the phonon bands, which reflect the hybridization of the plasmons with the phonons. The 187.4 meV of the static reference lies inside the upper phonon band of the hBN model (ε‖≈−3.69 + 0.159*i*) and therefore cannot be used as the prediction for an hBN stack. With the energy-dependent kernel, the main layer-odd and layer-even peaks at *q* = 0.2 ${\mathrm{nm}}^{-1}$ appear at 214.0 and 409.3 meV.

Figure 6c shows the layer-odd charge spectra of three hBN structures at *q* = 0.2 ${\mathrm{nm}}^{-1}$. The gapless 2 nm structure peaks at 214.0 meV. With 0.3 nm gaps above and below and 1.4 nm of hBN, the peak moves to 250.5 meV, a blue shift of 36.5 meV caused by the redistribution of dielectric material in the dynamic Coulomb kernel. The asymmetric structure, with gaps of 0.1 and 0.4 nm and outer dielectric constants of 1 and 4, peaks at 256.8 meV, where layer-parity mixing accompanies the energy shift. The energy dependence of the kernel changes the denominator of Equation (7) but not the sign structure of its numerator. Accordingly, the total-spin peak near 214.0 meV still appears for the opposite domain and that near 409.3 meV for the same domain (Figure S3), so the selection rule survives the phonon response and the dielectric loss. Close to a dielectric resonance, a peak should be identified by its energy, layer parity, and spectral weight together.

Top–bottom asymmetry mixes the layer-odd and layer-even channels and gives the dark branch a small weight. Figure 6d shows the ratio *R* of Equation (20) along the path of Equation (19). *R* is zero at the symmetric point, reaches 2.04% at |λ| = 1, is invariant under exchange of the top and bottom environments (λ → −λ), and grows approximately quadratically in the weakly asymmetric regime. Parity mixing gives the dark-branch vertex combination a first-order amplitude, whereas the spin response is formed from the product of two vertices and is therefore second order. Even at the end of the scan, the projection area of the former dark branch is only about 2% of that of the opposite-domain bright branch. Increasing the common electronic broadening η from 0.5 to 5 and 20 meV gives end-point ratios of 2.043%, 2.046% and 2.052%. Since η broadens both branches alike and *R* compares electronic branch corrections in the same window, its size is set by the structural asymmetry, and the bright–dark assignment is retained although the peaks become lower and wider (Figure S3). The environment also determines how an external source couples to the two layers: for a source 1 nm above the upper layer, the loss areas for the two bias polarities ($\mu_{1}$ = ±25 meV, $\mu_{2}$ = −$\mu_{1}$) in the 195–320 meV window differ by a factor of about 1.23 in the symmetric structure (Figure S4). The hBN environment therefore moves the plasmons through the denominator and leaves the domain selection, which is set by the numerator, intact.

*Other sources of layer asymmetry.* The dielectric environment is not the only way to break the layer symmetry. We tested unequal fillings and unequal broadenings in the gapless symmetric hBN structure at *q* = 0.2 nm${\mathrm{nm}}^{-1}$, with layer 1 fixed at $\mu_{1}$ = 0 and $\eta_{1}$ = 0.5 meV, and *R* was evaluated with Equation (20) (Section S10). For $\mu_{2}$ = δμ, *R* is 3.8 × $10^{-4}$% at δμ = 2 meV, 9.2 × $10^{-3}$% at 10 meV and 0.20% at 50 meV, and it follows a power law with an exponent of 1.95–1.96, the quadratic dependence expected for a perturbation of first-order amplitude. The layer-odd and layer-even peaks shift down with δμ, from 214.0 and 409.3 meV to 210.3 and 402.0 meV at δμ = 50 meV. For unequal broadenings $\eta_{2}$ = 2, 5, 10 and 20 times $\eta_{1}$, the magnitude of *R* is 5.3 × $10^{-5}$%, 8.5 × $10^{-4}$%, 4.3 × $10^{-3}$% and 1.9 × $10^{-2}$%, with an exponent of 1.99 in $\eta_{2}$/$\eta_{1}$ − 1, and the peaks do not shift beyond the energy step of 0.25 meV. In this case, the branch correction is negative, so that *R* is a signed correction and not an absorption ratio. The combination δμ = 10 meV and $\eta_{2}$ = 5$\eta_{1}$ gives 7.3 × $10^{-3}$%. In all cases *R* stays about ten times or more below the 2.04% of the structural asymmetry at |λ| = 1, so these electronic asymmetries do not change the bright–dark assignment of the branches.

### 3.5. Experimental Readout and Conditions of Validity

The plasmons described here lie in the mid-infrared and carry a large in-plane momentum (Table 2). The 187.4 meV mode corresponds to a free-space wavelength of about 6.62 µm, and the 214.0–256.8 meV peaks of Figure 6 correspond to 5.79–4.83 µm. A wave vector of *q* = 0.2 ${\mathrm{nm}}^{-1}$ corresponds to an in-plane mode wavelength of 31.4 nm, about 211 times the photon momentum at the same energy, so free-space light cannot excite these modes directly. Momentum-resolved electron energy-loss spectroscopy (EELS) and near-field coupling are the natural probes of the charge spectra. Photocurrent nanoscopy and mid-infrared scattering-type near-field microscopy have already been used for acoustic plasmons in graphene [12,13], and resonant coupling structures can bridge free-space excitation and large-momentum modes [14].

*Which observable distinguishes the two domain configurations.* These probes couple to the electron density. In the present model, the charge spectra of all four domain configurations are identical (Section 3.1), and the equality follows from the symmetry relations of Section 2.3 and not from a numerical coincidence. A charge-sensitive measurement therefore cannot distinguish the same-domain from the opposite-domain stack, and the domain selection of Section 3.1 is a property of the spin channel only. The observable that distinguishes the configurations is the total-spin weight at the two plasmon energies: the acoustic plasmon carries total-spin weight in the opposite-domain stack and the optical plasmon in the same-domain stack. Its measurement requires a spin-sensitive detection of the loss, for which spin-resolved electron energy loss has an experimental precedent [28]. We do not model the coupling of a probe to the spin, so the size of a spin-resolved signal in a given scattering geometry lies outside the present model. The domain configuration of a stack can, in addition, be determined independently by magnetic imaging of the layers, as performed for MnTe [25], so that the plasmon measurement tests the predicted spin weights instead of being used to infer the configuration.

*Layer-difference spin.* The layer-difference spin separates interlayer cancelation from the diagonal node, but it requires that the probe weights the two layers differently. For a near-field probe above the stack, the weight of the lower layer is reduced by approximately $e^{-qd}$, which is 0.67 for *q* = 0.2 ${\mathrm{nm}}^{-1}$ and *d* = 2 nm, so that the two layers are only weakly distinguished. With the weights (1, $e^{-qd}$), the same-domain acoustic plasmon, which has no total-spin weight for equal weights, acquires a spin signal of relative intensity $\tanh^{2}\left(qd/2\right)$, that is 3.9% at *q* = 0.2 ${\mathrm{nm}}^{-1}$ and *d* = 2 nm, and between 0.06% and 21% for *q* = 0.05–0.2 ${\mathrm{nm}}^{-1}$ and *d* = 1–5 nm (Section S16). Reconstructing the two layer amplitudes from two readings, one from each side with the weights (1, $e^{-qd}$) and ($e^{-qd}$, 1), requires the inversion of a matrix with condition number (1 + $e^{-qd}$)/(1 −$e^{-qd}$), which is 5.1 at *q* = 0.2 ${nm}^{-1}$ and *d* = 2 nm and 20 at *q* = 0.05 ${nm}^{-1}$. Independent errors of ±5% in the four weights then give errors of the layer difference of up to 25% and 100% for these two cases, whereas a calibration error of each layer that is the same in both readings leaves an error of 5%. We know of no established measurement geometry that provides the layer-resolved spin weights independently. The layer-difference spin is therefore a theoretical diagnostic that identifies the origin of a missing spin peak, and not an experimentally available readout. A source above the stack couples unequally to the two layers, as illustrated by the layer-bias calculation of Figure S4, which may offer partial layer selectivity.

*Conditions of validity.* The spacer must suppress interlayer tunneling so that the coupling remains Coulombic. The tunnel current through hBN decreases exponentially with the number of layers and is measurable down to a single layer [29], so a spacer of 1.4–2 nm, which corresponds to about 4–6 hBN layers, is expected to suppress tunneling. We have not calculated the tunneling amplitude between ${\mathrm{RbV}}_{2}$${\mathrm{Te}}_{2}$O layers, and this expectation should be checked for the actual stack. The relative domain of the two layers must also be settable. Domain patterning and field-assisted thermal cycling have been demonstrated in MnTe [25], but this does not establish equivalent control in ${\mathrm{Rb}}_{1-\delta }$$V_{2}$${\mathrm{Te}}_{2}$O, whose magnetic structure has only recently been determined [6], and a relative-domain configuration of two such layers has not been demonstrated. If the domains are larger than the plasmon wavelength (31 nm at *q* = 0.2 ${\mathrm{nm}}^{-1}$) and the probed area contains an area fraction *f* of opposite-domain regions, the measured total-spin weights are the corresponding weighted averages of the two limiting cases, namely *f* for the acoustic and 1 − *f* for the optical plasmon, so that incomplete alignment lowers the contrast but does not shift the energies. For domains smaller than the plasmon wavelength, the response is no longer a sum of intensities and requires a separate treatment.

The calculation holds the magnetic order and the band parameters fixed and includes the temperature only through the Fermi function at 300 K. The transition temperature reported for the G-type order of ${\mathrm{Rb}}_{1-\delta }$$V_{2}$${\mathrm{Te}}_{2}$O is 337 K [6], so that at 300 K the order is established, but its magnitude may be reduced compared with low temperatures. A smaller spin splitting would reduce the absolute spin signal, whereas the domain selection, which depends only on the symmetry relations of Section 2.3, is retained as long as the order persists. Whether Rb deficiency, carrier doping, or the hBN interface change the magnetic order or the band parameters enough to alter the plasmon energies is not included in the model. We assume that they do not change the symmetry, and the energies should be taken as model values (Section 3.6). The spin signal is the spin projection of the charge plasmons at a fixed magnetic order, and collective modes of the spin channel itself, which require spin-channel interactions [20,21], are not captured. The domain selection is insensitive to the electronic broadening and to top–bottom asymmetry beyond the 2% leakage of Section 3.4, whereas the plasmon energies depend on the interfacial gaps and dielectric constants (Figure 6c), which therefore have to be known when comparing with a measured peak. The filling scan of Section 3.3 spans a density change of about 2.18 × $10^{14}$${\mathrm{cm}}^{-2}$, which is large for conventional electrostatic gating and would require strong, for example, ionic gating that may also change the structure, the disorder, the screening and the magnetic order. The fixed-Hamiltonian result is therefore the intrinsic trend of the model and not a directly achievable tuning range. The moderate-doping calculation of Section 3.3 shows that a change of 1 × $10^{14}$${\mathrm{cm}}^{-2}$ softens the acoustic plasmon by 6.4%.

### 3.6. Scope, Approximations and Outlook

The calculations simplify the problem in four respects. First, the electronic structure is a four-band tight-binding model with the parameters of Ref. [7], which were not refitted. A minimal basis describes the static ground-state bands near the Fermi level well, but the dynamic response at energies of several hundred meV also involves transitions to bands outside the four-band subspace, and the density vertex uses the point-orbital approximation (Section 2.2). The plasmon energies are therefore model values with limited quantitative accuracy. What we expect to be robust is the symmetry structure of the spin signal (Section 2.3) and the orbital mechanism of the filling dependence (Section 3.3). We tested this with 22 modifications of the Hamiltonian that preserve the symmetry relation of Section 2.3: hoppings scaled by 0.8–1.2, the spin–orbit coupling scaled by 0–10, the *xy* on-site energy shifted by ±50 and ±100 meV, and ten random variations in the hoppings within ±10% (Section S14). In all cases, the symmetry relation holds to $10^{-15}$ eV, the mixed-vertex amplitudes at φ = $45^{\circ }$ vanish to 5.5 × $10^{-15}$, and the same-domain acoustic area stays below 6 × $10^{-4}$ of the opposite-domain one. The energies depend on the following parameters: at μ = 0, the acoustic energy lies between 151.6 and 223.2 meV, the optical energy between 334.2 and 492.0 meV, and $P^{2}$ between 0.46 and 0.71. The softening between μ = 0 and 0.2 eV persists in all cases and ranges from 9.4% to 17.7% at fixed μ and from 10.0% to 16.7% at fixed electron number, where the electron numbers of the original parameters at the two ends are kept. Multiplying the spin–orbit coupling by 10, changes the acoustic energy by less than 0.15%, so the charge plasmons are only weakly sensitive to it in this model. Second, the interaction is the Coulomb interaction in the density channel within the RPA, without vertex corrections and without an interaction in the spin channel. Third, the layers are coupled only by the Coulomb interaction, the magnetic order is static and uniform, and the filling is changed by a rigid shift of μ at fixed Hamiltonian. Fourth, the broadening η is a numerical parameter and does not represent disorder or phonon scattering of the electrons, and the only phonons included are those of the hBN spacer through its dielectric function.

The relation between μ and a real sample is not fixed by the model. Samples of this compound are Rb deficient, with δ ≈ 0.12 in the neutron-diffraction refinement of Ref. [6] and δ ≈ 0.2 with hole-dominated transport in Ref. [30]. We therefore use a relative mapping. If each Rb vacancy removes one electron from the $V_{2}$${\mathrm{Te}}_{2}$O layer and the cell contains one formula unit, δ = 0.12, 0.17 and 0.22, a range that spans these two values, correspond to density changes of −7.4, −10.5 and −13.6 × ${\mathrm{10}}^{13}$${\mathrm{cm}}^{-2}$ relative to the electron number of the model at μ = 0 (2.9886 electrons per cell at 300 K). This is an assumption. We adopt it because Ref. [7] does not define the electron number of the stoichiometric compound in the model, so that the model zero of μ cannot be identified with δ = 0. With this mapping, μ = −53, −73 and −91 meV, the stiffness $D_{xx}$ is larger by 9.6%, 13.6% and 17.5%, the acoustic RPA energy at *q* = 0.05 ${\mathrm{nm}}^{-1}$ is higher by 4.5%, 6.3% and 8.0%, and the relative spin amplitude is lower by 17%, 24% and 31% (Section S11). Rb-deficient samples then lie on the hole side of the scan of Figure 5, with a stiffer and less spin-projected acoustic plasmon than the model at μ = 0, and the softening of Section 3.3 corresponds to adding electrons to such a sample. Taking the nominal electron number of three per cell as the reference instead would shift all values of μ(δ) by an amount equivalent to 7 × $10^{12}$${cm}^{-2}$, which changes the acoustic energy by about 0.4% and is smaller than the differences between the three values of δ.

More accurate calculations are possible in the following order. A Wannier tight-binding model with the full set of V 3d and Te 5p orbitals, obtained from density-functional calculations for the magnetic structure determined in Ref. [6] and for the Rb content of the samples, would remove the restriction to four bands. The dynamic response could then be calculated from ab initio linear-response theory, including all interband transitions and the Bloch matrix elements of the density vertex, at the RPA level and with an exchange–correlation kernel. The Rb deficiency could be treated in a supercell or in the virtual-crystal approximation to relate μ to δ. An interaction in the spin channel, which is not included here, couples the spin projection to genuine spin collective modes [20,21] and is a separate problem; interlayer tunneling would further modify the layer-odd and layer-even modes. Each of these steps increases the computational cost considerably. The present model is intended to establish the symmetry structure and the trends that such calculations should test.

## 4. Conclusions

We have studied the charge plasmons of a Coulomb-coupled ${\mathrm{RbV}}_{2}$${\mathrm{Te}}_{2}$O bilayer and their projection onto the longitudinal spin, using a representative four-band tight-binding model with spin–orbit coupling and density-channel Coulomb interaction in the random-phase approximation. The main conclusions are as follows.

1. The relative magnetic domain controls which plasmon is visible in the total-spin channel and leaves the charge spectra unchanged, because it reverses the sign of the local mixed response of one layer.
2. The origin of a missing total-spin peak is identified by the layer-difference spin: interlayer cancelation leaves a complementary peak there, whereas the diagonal-direction node of the single-layer mixed response removes the peak from both spin channels.
3. Electron filling acts through the orbital composition of the current and tunes the energy of the acoustic plasmon together with its relative spin amplitude. The loss of low-energy *xy* current weight dominates the softening.
4. An hBN spacer shifts the plasmon energies but preserves the domain selection, with a second-order leakage of about 2% under layer asymmetry.

The charge spectra do not depend on the domain, so that the domain selection can be read out only with a spin-sensitive probe, and the layer-difference spin is a theoretical diagnostic. The symmetry-based results hold for models with the symmetry relations of Section 2.3, whereas the plasmon energies and their filling dependence are model values. These results provide guidance for designing spin-selective altermagnetic plasmonic nanostructures.

## Figures and Tables

**Table 1:** Collective spin projection for same and opposite domains in a symmetric, equally filled bilayer.

Relative Domain	Acoustic: Total Spin $S_{+}$	Acoustic: Layer-Difference Spin $S_{-}$	Optical: Total Spin $S_{+}$	Optical: Layer-Difference Spin $S_{-}$
Same (++)	dark	bright	bright	dark
Opposite (+−)	bright	dark	dark	bright

$S_{+}$ = $S_{1}$ + $S_{2}$ and $S_{-}$ = $S_{1}$−$S_{2}$. Bright and dark indicate that the charge branch has or lacks a collective spin projection; the complete spin spectrum also contains the bare-response background. The table applies to propagation directions in which the single-layer mixed response is finite.

**Table 2:** Energy and momentum scales of representative charge responses.

Structure or Filling	*q* (${\mathrm{nm}}^{-1}$)	*E* (meV)	Free-Space Wavelength **λ**_**0**_ (µm)	*q*/$q_{0}$
Static reference: acoustic	0.20	187.4	6.62	211
Static reference: optical	0.20	413.1	3.00	95.5
Dynamic hBN: layer-odd peak	0.20	214.0	5.79	184
Dynamic hBN: layer-even peak	0.20	409.3	3.03	96.4
Filling scan: μ = 0	0.05	50.15	24.7	197
Filling scan: μ = 0.2 eV	0.05	42.52	29.2	232

$q_{0}$ = 2π/$\lambda_{0}$ is the wave vector of a free-space photon of the same energy. The in-plane mode wavelengths for *q* = 0.20 and 0.05 ${\mathrm{nm}}^{-1}$ are 31.4 and 126 nm. The dynamic hBN entries refer to the gapless symmetric structure, and the filling scan corresponds to Figure 4d.

## Data Availability

The numerical data of all figures and the programs used for the calculations are openly available in Zenodo at https://doi.org/10.5281/zenodo.23291214.

## Supplementary Materials

The following supporting information can be downloaded at: https://www.mdpi.com/article/10.3390/nano16211430/s1, Sections S1–S16: derivations, symmetry arguments, numerical checks and additional calculations; Figure S1: Optical layer phases and layer-difference spin channels; Figure S2: Angular dependence of the relative spin projection; Figure S3: Domain selection in symmetric dynamic hBN at different common broadening; Figure S4: Layer-bias readout with a fixed upper source; Figure S5: Energy–momentum distribution of the bare electronic density response; Table S1: Charge-window areas of the full and intraband-only responses; Table S2: Numerical settings of the calculations; Table S3: Stiffness, relative spin amplitude and acoustic energy as functions of the chemical potential; Table S4: Orbital decomposition of the current stiffness and the spin-weighted stiffness; Table S5: Convergence of the main-text settings with respect to the finest common reference; Table S6: Largest relative deviation from the finest common reference as a function of the momentum grid and of the number of transfer-energy bins; Table S7: Edges of the bare particle–hole continua and positions of the plasmon peaks; Table S8: Peak widths of the plasmons; Table S9: Dark-branch ratio *R* for unequal layer fillings and broadenings in the gapless symmetric hBN structure; Table S10: Response for changes of the electron density; Table S11: Slope of the long-wavelength acoustic plasmon energy and position of the local curvature extremum; Table S12: Dependence on the relative twist of the second layer; Table S13: Sensitivity to symmetry-preserving parameter changes; Table S14: Integrated spectral weights of the spin and charge peaks; Table S15: Layer-weighted detection.

## Abbreviations

The following abbreviations are used in this manuscript:

hBN	hexagonal boron nitride
RPA	random-phase approximation
SOC	spin–orbit coupling
TO/LO	transverse/longitudinal optical
