# Supplementary material for: Domain-Selected Spin Projection and Orbital-Filling Softening of Charge Plasmons in a Model Altermagnetic RbV2Te2O Coulomb Bilayer
Source: Nanomaterials (Basel). 2026 Nov 5;16(21):1430. doi: 10.3390/nano16211430 (PMC13765314; doi:10.3390/nano16211430)
Supplement: Supplementary file 1 [file nanomaterials-16-01430-s001.zip › supplementary.pdf]

nanomaterials

Article

Supplementary Materials: Domain-Selected Spin Projection and Orbital-Filling Softening of Charge Plasmons in a Model Altermagnetic RbV_2Te_2O Coulomb Bilayer

Shun Zhou [1,2,*], Qi Zhang [1] and Fangwei Han [1,*]

1 School of Medical Information Engineering, Jining Medical University, Jining 272067, China
2 Robinson Research Institute, Victoria University of Wellington, Lower Hutt 5046, New Zealand
* Correspondence: zsphys@mail.jnmc.edu.cn (S.Z.); fwhan@mail.jnmc.edu.cn (F.H.)

Abstract

Supplementary information for the main manuscript: derivations, symmetry arguments, numerical checks and tabulated data.

Keywords: altermagnet; Coulomb bilayer; plasmon; random-phase approximation; hBN

Check for updates

Academic Editor: Martijn Wubs

Received: 11 October 2026
Revised: 1 November 2026
Accepted: 3 November 2026
Published: 5 November 2026

Copyright: © 2026 by the authors. Licensee MDPI, Basel, Switzerland. This article is an open access article distributed under the terms and conditions of the Creative Commons Attribution (CC BY) license.

This document supplies the derivations, checks and data behind the main text. Section S1 derives the layer-parity form of Equation (7) and completes the domain selection of Section 3.1 for the optical branch and the layer-difference readout. Section S2 gives the symmetry argument for the diagonal node of Section 3.2 and the long-wavelength relations of Sections 2.4 and 3.3. Sections S3 and S4 test the dynamic-hBN results of Section 3.4 against the electronic broadening and a one-sided source, and Section S6 describes the dynamic Coulomb kernel they rely on (Section 2.5). Section S5 quantifies the interband contribution and the 45° background discussed in Sections 3.2 and 3.3. Section S7 collects the numerical settings and the tabulated filling data (Sections 2.6 and 3.3). Reference numbers refer to the reference list of the main text.

S1. Layer-Parity Structure and Optical Layer Phases

S1.1. Layer-Parity Response and Domain Transformation

Equation (7) of the main text follows from the RPA response of Equation (4) once the two layers are grouped by parity. We write the density bare response of each layer as $P_N = \mathrm{diag}(p_1, p_2)$ and let $\mathbf{b} = (r_1, r_2)$ and $\mathbf{c} = (c_1, c_2)^T$ be the layer vectors of Π_{SN} and Π_{NS}. The Coulomb correction to the total spin is

$$\Delta\chi_{S_+S_+} = \mathbf{b}\, V_N\, (I - P_N V_N)^{-1}\, \mathbf{c}. \tag{S1}$$

For equal layer responses $p_1 = p_2 = p$ and a symmetric environment, $e_\pm = (1, \pm 1)^T/\sqrt{2}$ gives

$$\Delta\chi_{S_+S_+} = \sum_{\alpha=\pm} (\mathbf{b}\, e_\alpha)\, \frac{v_\alpha}{1 - p\, v_\alpha}\, (e_\alpha^T \mathbf{c}). \tag{S2}$$

The layer-difference spin corresponds to $\mathbf{b}_- = (r_1, -r_2)$ and $\mathbf{c}_- = (c_1, -c_2)^T$. Reversing the sign of the mixed response of one layer therefore exchanges the branch projections of the total and layer-difference spin, while the two charge denominators remain unchanged. In the dissipative response, the left and right mixed vertices enter as ordered products.

Under time reversal the density is even and the spin odd, and the two domains satisfy

$$\Pi^{-}_{ab}(\mathbf{q}, E) = \epsilon_a \epsilon_b \, \Pi^{+}_{ba}(-\mathbf{q}, E), \qquad \epsilon_N = 1, \, \epsilon_S = -1. \tag{S3}$$

Equation (S3) also exchanges the NS and SN indices. Along the x direction of Figure 2 of the main text, the inversion and directional symmetry of the model make the two mixed vertices equal, so that the opposite-domain transformation appears as a sign reversal. Reversing both domains leaves the autocorrelation spectra unchanged, so ++ and $--$, and $+-$ and $-+$, have equal intensities. Away from this direction the calculation keeps both ordered mixed responses.

S1.2. Optical Layer Phases and Complementary Layer-Difference Spectra

Figure S1 complements Figure 3a,b and Figure 2 of the main text. For an in-phase optical charge oscillation, the two layer spins add in the same domain and cancel in the opposite domain, and the layer-difference readout exchanges the two cases. The total and layer-difference spin of the acoustic branch are likewise complementary. Along the directional node the mixed responses of both layers vanish, so both layer combinations lose the collective projection. The mirror relation at the symmetric point follows from Equation (S2), and the layer-difference channel is used to establish whether a finite local signal remains when the total spin has no peak.

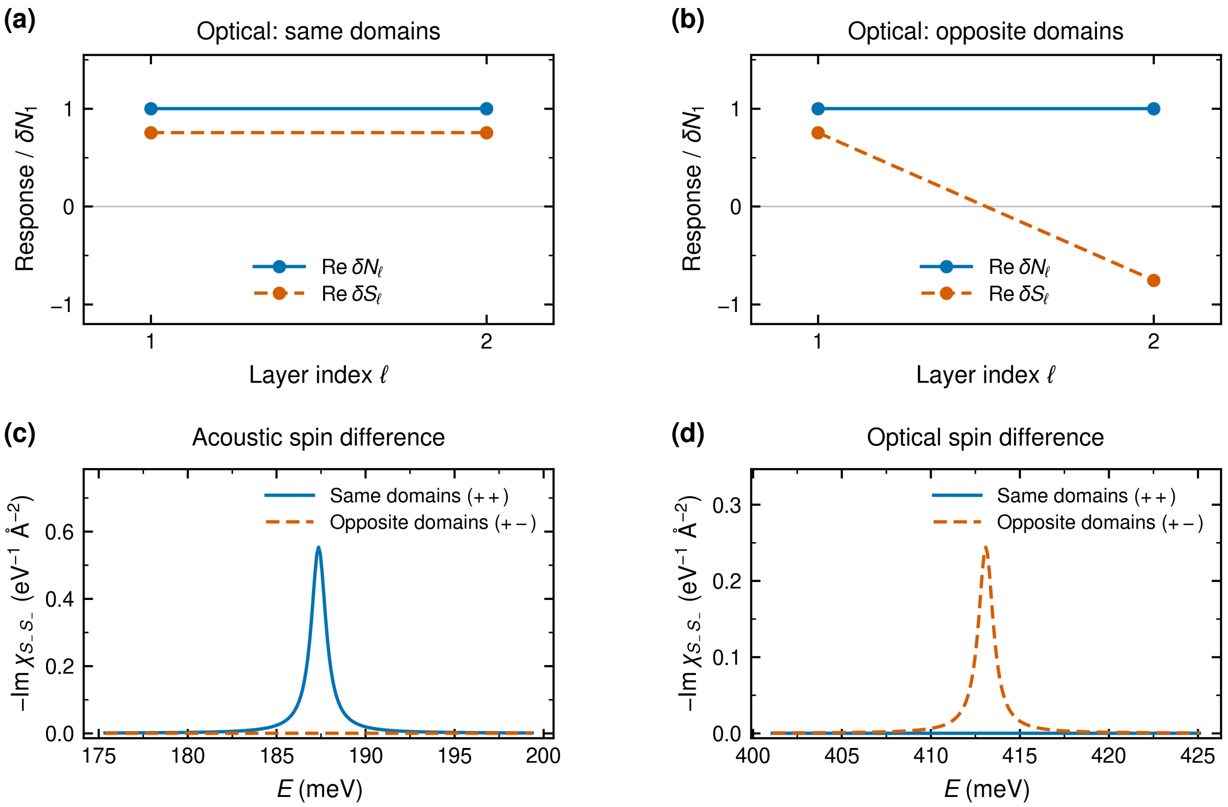

Figure S1. Optical layer phases and layer-difference spin channels. (**a**,**b**) Density-driven layer amplitudes at the optical response condition for same and opposite domains; (**c**,**d**) layer-difference spin spectra in the acoustic and optical windows. Settings as in Figure 2 of the main text; (**a**,**b**) use $\delta N_1 = 1$ and the lines connect the two discrete layer points.

The antiphase degree of freedom of the acoustic charge branch here is the layer density, whereas the antiphase partners of the spin-demon modes of single-layer altermagnets involve spin components [20,21]. The two cases can be told apart by their layer and spin-component structure.

https://doi.org/10.3390/nano16211430

S2. Diagonal Node and Spin-Weighted Stiffness

S2.1. Directional Node with SOC and 90° Correspondence

This section gives the symmetry argument behind the node of Section 3.2. In the four-orbital basis of the main text, the unitary matrix of the momentum exchange can be written explicitly as

$$U=\begin{pmatrix}0&0&i&0\\0&0&0&i\\1&0&0&0\\0&1&0&0\end{pmatrix}. \tag{S4}$$

With the atomic Bloch gauge and point-orbital vertices used here, the Hamiltonian satisfies

$$H(k_x,k_y)=U\,H(k_y,k_x)\,U^{\dagger},\qquad UNU^{\dagger}=N,\qquad USU^{\dagger}=-S. \tag{S5}$$

Exchanging $k_x \leftrightarrow k_y$ in the Brillouin-zone integral gives $\Pi_{SN}(q_x,q_y,E)=-\Pi_{SN}(q_y,q_x,E)$, and likewise for NS. For $q_x=q_y$ the mixed responses vanish, whereas the NN and SS autocorrelations remain finite. The node results from the pairwise cancellation of all momentum states and their matrix elements, and both SOC terms are contained in Equation (S5). Reconstructing the full SOC Hamiltonian at 40 random momentum points, the largest matrix-element deviation of Equation (S5) is 1.0×10^{-15} eV. Numerically, at φ = 45°, q = 0.2 nm^{-1} and E = 187.364 meV, the mixed response is $|\Pi_{\mathrm{SN}}|=2.0\times10^{-22}$, compared with $|\Pi_{\mathrm{NN}}|=2.64\times10^{-3}$ in the same units. The density-driven relative amplitude $\Pi_{\mathrm{SN}}/\Pi_{\mathrm{NN}}$ at the acoustic real-axis crossing is 0.7616 at φ = 0°, 0.6345 at 17°, ≈ 0 at 45°, −0.6345 at 73° and −0.7616 at 90°.

The opposite-domain response of this model also corresponds to that of the reference domain rotated by 90°. At φ = 0°, 17°, 45°, 73° and 90°, with a 256 × 256 grid, μ = 0, q = 0.2 nm^{-1}, E = 187.364 meV and η = 0.5 meV, the two full bare response matrices were compared directly. The largest difference relative to $|\Pi_{\mathrm{NN}}|$ is below 3×10^{-15}. This correspondence is used for orthogonal layer orientations in the Coulomb environment without tunneling and with isotropic in-plane interaction. An arbitrary twist angle requires the corresponding directional responses and structural kernels.

S2.2. Long-Wavelength Interpretation of the Spin-Weighted Stiffness

Here we derive the long-wavelength relations used in Sections 2.4 and 3.3 and compare them with the full response. In the low-q expansion of intraband vertices, the density vertex tends to 1 and the spin vertex to the eigenstate expectation value $s_{n\mathbf{k}}$. With $u_i=\partial E_{n\mathbf{k}}/\partial(k_i a)$, the inertial leading terms of the intraband response are

$$\Pi_{NN}\simeq\frac{q_A^2 D_{\phi\phi}}{E^2},\qquad \Pi_{SN}\simeq\frac{q_A^2 D^s_{\phi\phi}}{E^2}, \tag{S6}$$

with

$$D^s_{ij}=\frac{1}{N_k^2}\sum_{n\mathbf{k}}\left(-\frac{\partial f}{\partial E_{n\mathbf{k}}}\right)s_{n\mathbf{k}}\,u_i\,u_j. \tag{S7}$$

Equation (S6) keeps the first nonzero term of an expansion in $q_A a|u|/E$; the full finite-q intraband response contains higher-order band and vertex information. SOC makes the longitudinal spin expectation value of the eigenstates deviate from a pure spin label, and Equation (S7) weights accordingly, so the response is not equated with two strictly independent spin fluids.

https://doi.org/10.3390/nano16211430

On a 512 × 512 grid the model gives $D_{xx} = D_{yy}$, $D_{xy} = 0$, $D^s_{xx} = -D^s_{yy}$ and $D^s_{xy} = 0$ within numerical precision. With $P = D^s_{xx}/D_{xx}$,

$$\frac{\Pi_{SN}}{\Pi_{NN}} \simeq P\cos(2\phi), \qquad \left|\frac{\Pi_{SN}}{\Pi_{NN}}\right|^2 \simeq P^2\cos^2(2\phi). \tag{S8}$$

At μ = 0, $D_{xx} = 0.2237$ eV, $D^s_{xx} = 0.1683$ eV and P = 0.7524. At the acoustic real-axis condition for q = 0.2 nm^{-1} the amplitude ratio of the full response is about 0.7616 − 5.08 × 10^{-5} i. Figure S2 compares the finite-q result with Equation (S8): the directional node is the same, and the amplitude carries finite-wave-vector corrections. The squared modulus measures the density-driven relative spin amplitude squared; the peak of the full spin spectrum also depends on the spectral weight of the charge branch, the bare background and the broadening.

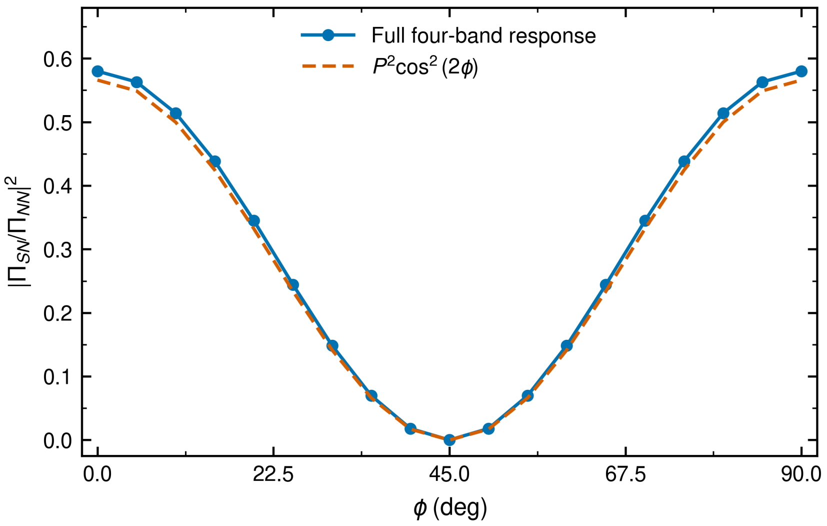

Figure S2. Angular dependence of the relative spin projection. μ = 0, q = 0.2 nm^{-1}, κ = 4, d = 2 nm, T = 300 K. Blue dots: squared modulus of the ratio at the acoustic real-axis condition of the full response in each direction; orange dashed line: leading long-wavelength term of Equation (S8). The full response uses a 256 × 256 grid and η = 0.5 meV, and the stiffness a 512 × 512 grid.

S3. Domain Selection and Asymmetric Leakage at Different Common Broadening

Section 3.4 states that neither the bright–dark assignment nor the leakage ratio depends on the electronic broadening. Figure S3 shows this by comparing η = 0.5, 5 and 20 meV for gapless 2 nm hBN with outer dielectric constants of 4, μ = 0, q = 0.2 nm^{-1}, φ = 0° and T = 300 K. The calculation uses a 384 × 384 grid, a 32,768-bin histogram, an energy step of 0.25 meV and the total-spin probe $(S_1 + S_2)/\sqrt{2}$. A larger η broadens the peaks and lowers their height; in both main charge windows the bright-branch assignment (acoustic-type for the opposite domain, optical-type for the same domain) is retained.

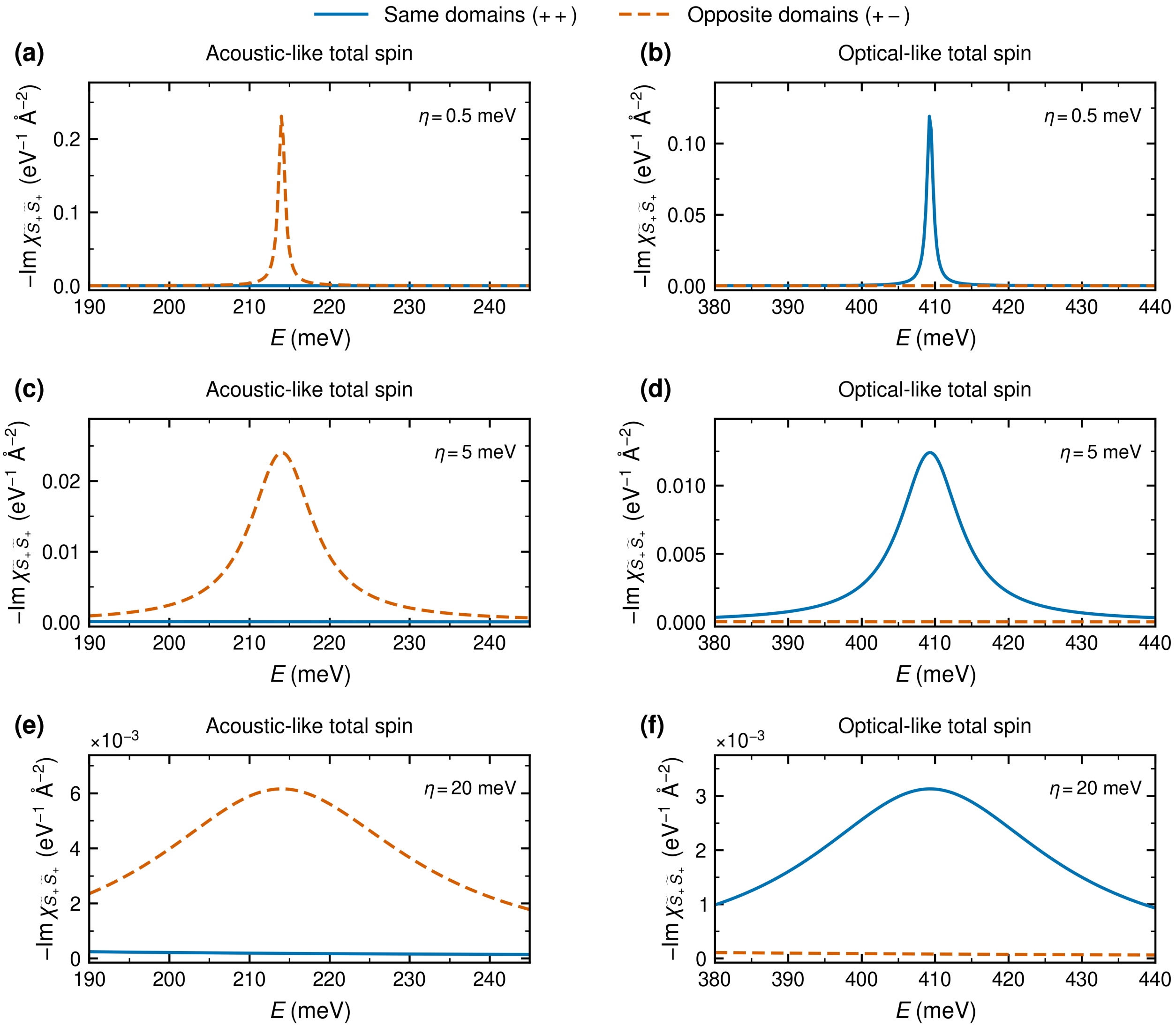

Figure S3. Domain selection in symmetric dynamic hBN at different common broadening. (**a**,**b**) η = 0.5 meV; (**c**,**d**) 5 meV; (**e**,**f**) 20 meV. The left column shows the main layer-odd peak window and the right column the main layer-even peak window; blue solid lines: same domain; orange dashed lines: opposite domain. The vertical axis is the unit-normalized total-spin spectrum.

Along the asymmetric path of Equation (19) of the main text, the area ratio of Equation (20) at $\lambda = 1$ is 2.043%, 2.046% and 2.052% for the three values of η. The ratio uses the electronic correction of the layer-odd-type channel and the fixed window of 195–320 meV. The common η is a phenomenological broadening for sensitivity analysis, and it has not been calibrated to the scattering rate of this material. At the symmetric point the cancellation is controlled by the vertex combination, and the finite leakage along the specified structural path varies little in this scan.

S4. Layer-Bias Readout with a Fixed One-Sided Source

Section 3.4 mentions the response to a fixed one-sided source; here we give the formalism. The source stays at a height $h_s = 1$ nm above the upper layer and $\mu_2 = -\mu_1$. Let V_N be the Green's matrix between the two charge planes and $p_i = \Pi^{(i)}_{NN}$. The density denominator is $F = (1 - V_{11}p_1)(1 - V_{22}p_2) - V_{12}V_{21}p_1p_2$, and layer exchange gives

$$F(p_1, p_2) - F(p_2, p_1) = -(V_{11} - V_{22})(p_1 - p_2). \quad \text{(S9)}$$

For a symmetric environment $V_{11} = V_{22}$, so the mode denominator is unchanged when the two layer fillings are exchanged, whereas the fixed upper source gives the two layers different spatial weights.

Let A be the interface admittance matrix defined in Section S6, with charge-plane indices i_1, i_2 and upper-boundary index 0. The source transfer vector is $u_k = 2\kappa_t e^{-qh_s}(A^{-1})_{i_k 0}$ and the upper reference Coulomb quantity is $v_t = 2\pi e^2/(\kappa_t q_A)$. The full source Green's function divided by this reference is

$$\frac{G_{ss}}{v_t} = 1 + \left[2\kappa_t (A^{-1})_{00} - 1\right] e^{-2qh_s} + v_t\, u^T \chi_{NN} u. \tag{S10}$$

Here χ_{NN} is the full density response matrix of the two layers, and $e^2 = 14.40$ eV Å. The source loss is $\mathcal{L}_s = -\mathrm{Im}(G_{ss}/v_t)$. The reciprocal Green's function uses the transposed contraction $u^T \chi_{NN} u$; the first two terms are the environmental background and the last is the electronic correction. Figure S4 plots the full source loss. The bias contrast is defined as

$$R_{\mathrm{bias}} = \frac{\int_{195}^{320} \mathcal{L}_s(E; \mu_1 = +25\ \mathrm{meV})\, dE}{\int_{195}^{320} \mathcal{L}_s(E; \mu_1 = -25\ \mathrm{meV})\, dE}, \tag{S11}$$

with energies and limits in meV. The gapless symmetric structure gives $R_{\mathrm{bias}} = 1.229$ with both peaks near 214.0 meV; the asymmetric structure of Figure 6c of the main text gives 1.312, with positive and negative bias peaks at about 258.0 and 255.3 meV. The same structure at equal filling peaks at 256.8 meV. The ratio compares the source loss of two filling configurations, in which the source weight of the two layers is already contained in the response through Equation (S10).

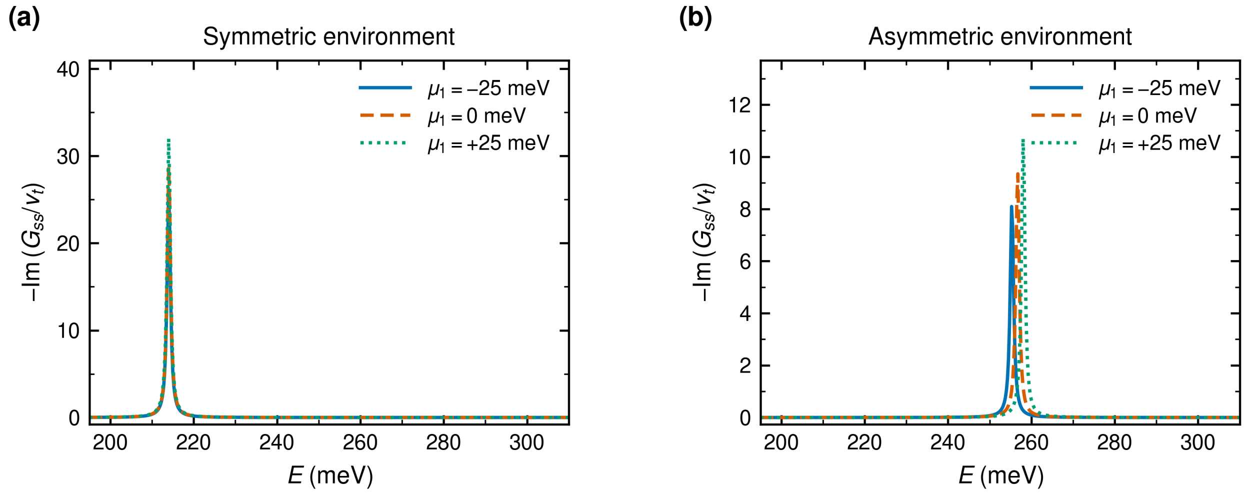

Figure S4. Layer-bias readout with a fixed upper source. Source height 1 nm, $\mu_2 = -\mu_1$. (**a**) Gapless symmetric structure; (**b**) asymmetric structure of Figure 6c. The full source response is divided by v_t and shown from 195 to 310 meV; the integration window is 195–320 meV. $q = 0.2$ nm^{-1}, $T = 300$ K, $\eta = 0.5$ meV; 512 × 512 grid and a 65,536-bin transfer-energy histogram.

S5. Interband Contributions and the 45° Bare Background

S5.1. Representative Charge Windows

This section supports the statement of Section 3.3 that the charge response is dominated by intraband transitions. The spectra of the main text keep all intraband and interband transitions of the four-band model. Table S1 compares the full response with a response that keeps only $n = m$ transitions, for static $\kappa = 4$ and $d = 2$ nm, a 256 × 256 grid, a 32,768-bin

transfer-energy histogram, η = 0.5 meV and an energy step of 0.25 meV. The window extends 12 meV on each side of the full-response charge peak, and the same charge probe is used for both spectra.

Table S1. Charge-window areas of the full and intraband-only responses.

μ (eV)	q (nm^{-1})	Charge branch	Full-response peak (meV)	Intraband peak (meV)	Full/intraband area
0	0.20	acoustic	187.25	187.25	0.999994
0	0.20	optical	413.00	413.00	0.999777
0.20	0.05	acoustic	42.50	42.50	1.000000
0.20	0.05	optical	185.25	185.25	0.999998
0.20	0.20	acoustic	159.00	159.00	0.999999
0.20	0.20	optical	348.75	348.75	0.999987
0	0.05	acoustic	50.25	50.25	1.000000
0	0.05	optical	219.75	219.75	0.999989

Peak positions are the maxima on the 0.25 meV sampling grid, and the last two digits identify the sampled point. The area ratios are given to six decimals to resolve the small interband correction; the original precision is kept in the CSV files.

In all tested windows the charge areas differ by less than 0.03%, so they are determined mainly by the intraband response. A separate comparison at μ = 0 and q = 0.2 nm^{-1} gave an interband correction of the acoustic real-axis crossing of about 0.000117 meV, and a full-to-intraband area ratio of the spin window of about 0.999976; this spin comparison uses a different definition from the charge-area ratio of Table S1. The intraband dominance supports the stiffness interpretation, while the finite-q Lindhard response still contains the full band dispersion and vertex information.

S5.2. Origin of the Diagonal-Direction Background

This section identifies the origin of the finite spin background that remains at the node in Section 3.2. For φ = 45°, μ = 0, q = 0.2 nm^{-1} and η = 0.5 meV, Equation (3) of the main text was summed directly on a 384 × 384 grid. At E = 186 meV, $-\mathrm{Im}\,\Pi_{\mathrm{NN}}$ = 1.4932 × 10^{-5} and $-\mathrm{Im}\,\Pi_{\mathrm{SS}}$ = 1.4964 × 10^{-5} eV^{-1} Å^{-2}; the intraband spin term is 1.4930 × 10^{-5} and the interband term 3.45 × 10^{-8} eV^{-1} Å^{-2}. The bare spin and density losses differ by about 0.22%, and the interband term is about 0.23% of the bare spin loss. With SOC switched off both quantities are 1.4932 × 10^{-5} eV^{-1} Å^{-2}. The background is therefore mainly the intraband response at finite η, and the directional node removes the collective enhancement in the mixed channel.

S6. Dynamic Dielectric Kernel and Energy–Momentum Spectra

This section describes the construction of the dynamic Coulomb kernel used in Section 2.5. Each dielectric interface is taken as a node. A uniaxial layer of thickness d_l with in-plane and out-of-plane dielectric components $\epsilon_{t,l}$ and $\epsilon_{z,l}$ defines $r_l = \sqrt{\epsilon_{t,l}/\epsilon_{z,l}}$ and $Y_l = \epsilon_{z,l} r_l$. Its admittance contribution to the neighboring nodes is

$$A_l = Y_l \begin{pmatrix} \coth(q d_l r_l) & -\mathrm{csch}(q d_l r_l) \\ -\mathrm{csch}(q d_l r_l) & \coth(q d_l r_l) \end{pmatrix}. \quad \text{(S12)}$$

The matrix A is assembled from the contributions of adjacent nodes of all layers, with κ_t and κ_b added to the diagonal elements of the top and bottom boundaries. An isotropic gap has $r_l = 1$ and $Y_l = \epsilon_l$. For charge-plane indices i and j the Coulomb kernel is

$$V_{N,ij} = \frac{4\pi e^2}{q_A}\,(A^{-1})_{ij}. \quad \text{(S13)}$$

For a gapless structure with symmetric outer media, with $r = \sqrt{\epsilon_t/\epsilon_z}$ and $Y = \epsilon_z r$, the layer-even and layer-odd kernels are

$$v_+ = \frac{4\pi e^2/q_A}{\kappa + Y\tanh(qdr/2)}, \qquad v_- = \frac{4\pi e^2/q_A}{\kappa + Y\coth(qdr/2)}. \tag{S14}$$

For the isotropic case $\epsilon_t = \epsilon_z = \kappa$, Equation (S14) recovers $v_\pm$ of Equation (5) of the main text. The dynamic hBN uses the Lorentz parameters of Equation (18) of the main text; the electronic response is evaluated at $E + i\eta$ and the dielectric response at the real energy E, where it keeps its own phonon damping.

Figure 6a,b uses 61 uniformly spaced q points (0.03–0.25 nm^{-1}) and 1,521 energy points (80–460 meV), with a 192 × 192 grid, a 16,384-bin histogram and η = 0.5 meV at each q. All local spectral peaks are provided as CSV files. The near-horizontal spectral structures in the figures arise from the joint response of the dispersive medium and electronic screening; tracking the branches near the phonon bands and quantifying their splitting would require a finer local scan.

Figure S5 shows the bare electronic density loss on the same energy–momentum grid. This finite-temperature, finite-broadening spectrum allows the charge resonances to be compared with the electronic background; its color scale does not define the boundary of a strict zero-temperature particle–hole continuum.

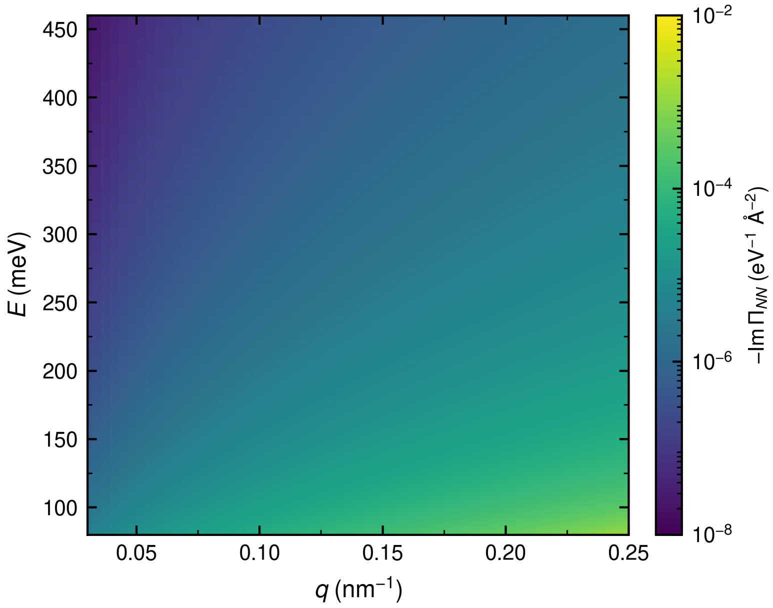

Figure S5. Energy–momentum distribution of the bare electronic density response. μ = 0, φ = 0°, T = 300 K, η = 0.5 meV, with the grid and sampling of Figure 6a,b. The color scale is the base-10 logarithm of the bare density loss in eV^{-1} Å^{-2}.

S7. Numerical Settings and Tabulated Data

Table S2 lists the settings of every calculation in the main text (Section 2.6), and Tables S3 and S4 give the filling data behind Figures 4 and 5 (Section 3.3). The convergence of the numerical settings is documented in Section S8, the continuum edges and peak widths in Section S9, the layer asymmetry from unequal filling and broadening in Section S10, the electron-number mapping and moderate density changes in Section S11, the Fermi-surface topology in Section S12, the relative twist in Section S13, the parameter sensitivity in Section S14, the absolute spectral weights in Section S15 and the layer-weighted detection in Section S16.

Table S2. Numerical settings of the calculations.

Calculation (figure)	Momentum grid	Transfer-energy histogram bins	q (nm^{-1})	η (meV)
Static domain and direction spectra (Figures 2, 3, S1)	384 × 384	32,768	0.2	0.5
Orbital occupation and stiffness (Figures 4a–c, 5a)	512 × 512	-	-	-
Filling-dependent acoustic RPA energy (Figure 4d)	256 × 256	-	0.05	1
Filling and angular projection (Figures 5b,c, S2)	256 × 256	32,768	0.05, 0.2	0.5
Dynamic hBN energy–momentum spectra (Figure 6a,b)	192 × 192	16,384	0.03–0.25 (61 points)	0.5
Dynamic hBN peaks and one-sided source (Figures 6c, S4)	512 × 512	65,536	0.2	0.5
Dynamic domain and broadening comparison (Figures 6d, S3)	384 × 384	32,768	0.2	0.5, 5, 20

All calculations use a fixed magnetic order and a 300 K Fermi distribution. Static spectra use a window of 12 meV on each side of the real-axis crossing with an energy step of 0.1 meV; the energy kernel of Figure 4c is accumulated in 2 meV bins and merged into 10 meV bins for display.

Table S3. Stiffness, relative spin amplitude and acoustic energy as functions of the chemical potential (φ = 0°, d = 2 nm, κ = 4, T = 300 K, η = 0.5 meV).

μ (eV)	n	D_{xx} (eV)	D^s_{xx} (eV)	P	P^2	E_1 (meV)	A_1	E_2 (meV)	A_2
−0.20	2.428	0.3120	0.1420	0.4550	0.2070	58.90	0.218	219.7	0.219
−0.15	2.589	0.2909	0.1533	0.5270	0.2777	56.90	0.289	212.3	0.291
−0.10	2.743	0.2672	0.1629	0.6095	0.3715	54.62	0.384	203.9	0.385
−0.05	2.877	0.2436	0.1673	0.6870	0.4719	52.26	0.484	195.1	0.486
+0.00	2.989	0.2237	0.1683	0.7524	0.5660	50.17	0.578	187.4	0.580
+0.05	3.086	0.2071	0.1668	0.8055	0.6489	48.34	0.660	180.6	0.662
+0.10	3.176	0.1901	0.1629	0.8571	0.7346	46.39	0.745	173.3	0.746
+0.15	3.262	0.1728	0.1571	0.9093	0.8269	44.32	0.835	165.6	0.836
+0.20	3.340	0.1589	0.1507	0.9479	0.8985	42.54	0.904	159.0	0.904

n: occupation per unit cell; E_1 and E_2: acoustic real-axis crossing at q = 0.05 and 0.2 nm^{-1}; A_1 and A_2: $|\Pi_{SN}/\Pi_{NN}|^2$ at the same crossings. The acoustic energies of this table use η = 0.5 meV; Figure 4d uses η = 1 meV, which gives 50.15 and 42.52 meV at q = 0.05 nm^{-1} for μ = 0 and 0.2 eV.

Table S4. Orbital decomposition of the current stiffness and the spin-weighted stiffness.

μ (eV)	Orbital weight	D_{xx} (eV)	D^s_{xx} (eV)	D^s_{xx}/D_{xx}
0.0	xy	0.05709	0.02096	0.367
0.0	xz/yz	0.16661	0.14734	0.884
0.2	xy	0.00278	0.00098	0.353
0.2	xz/yz	0.15615	0.14968	0.959

S8. Numerical Convergence

The momentum grid $N \times N$ and the number of transfer-energy bins were varied independently for the quantities that carry the main results. The grid was varied over N = 128, 192, 256, 384, 512, 768 and 1024 at 32,768 bins, and the number of bins over 8192, 16,384, 32,768, 65,536 and 131,072 at N = 384. The transfer-energy range is 0–3 eV, so that the bin width is 3000/(number of bins) meV, which is 0.092 meV for 32,768 bins. All tests use T = 300 K, φ = 0°, d = 2 nm and a static background dielectric constant κ = 4; the

static spectra and the leakage ratio R use η = 0.5 meV, and the filling calculations use η = 1 meV. Table S5 compares the settings of the main text (N = 384, 32,768 bins) with the finest common reference (N = 1024, 131,072 bins).

Table S5. Convergence of the main-text settings (N = 384, 32,768 bins) with respect to the finest common reference (N = 1024, 131,072 bins).

Quantity	η (meV)	N = 384, 32,768 bins	Reference	Relative deviation (%)
Acoustic energy, static, $q = 0.2\ \mathrm{nm}^{-1}$ (meV)	0.5	187.363947	187.363841	5.7×10^{-5}
Optical energy, static, $q = 0.2\ \mathrm{nm}^{-1}$ (meV)	0.5	413.092442	413.092227	5.2×10^{-5}
Relative spin amplitude $\lvert\Pi_{SN}/\Pi_{NN}\rvert^2$, static, $q = 0.2\ \mathrm{nm}^{-1}$	0.5	0.579987	0.579986	9.5×10^{-5}
Leakage ratio $R(\lambda = 1)$ (%)	0.5	2.04343	2.04343	8.0×10^{-6}
Acoustic energy, $\mu = 0$, $q = 0.05\ \mathrm{nm}^{-1}$ (meV)	1	50.1461	50.1460	1.3×10^{-4}
Acoustic energy, $\mu = 0.2$ eV, $q = 0.05\ \mathrm{nm}^{-1}$ (meV)	1	42.5162	42.5163	1.7×10^{-4}
Relative spin amplitude $\lvert\Pi_{SN}/\Pi_{NN}\rvert^2$, $\mu = 0.2$ eV, $q = 0.05\ \mathrm{nm}^{-1}$	1	0.903684	0.903684	1.9×10^{-5}

The largest deviation of the main-text settings is 1.7×10^{-4}%, and all quantities of Table S5 deviate by less than 0.1% from the finest reference. Table S6 shows that the deviations stay below 0.002% for every grid and every bin number tested, including the coarsest ones (128 × 128 and 8192 bins), so that the criterion of 0.1% is met by all settings; the bin number has a slightly larger effect than the grid (largest deviation 1.1×10^{-3}% at 8192 bins), and the leakage ratio R converges faster than the other quantities. Reducing η from 0.5 meV to 0.25 meV (from 1 meV to 0.25 meV for the filling calculations) shifts the static acoustic and optical energies by +0.0016 and +0.0006 meV, the filling-calculation energies at $\mu = 0$ and 0.2 eV by +0.030 and +0.036 meV (0.06% and 0.08%), and R from 2.0434% to 2.0433%. The test examines the discretization of the Brillouin zone and of the transfer energy; η itself is a numerical broadening and not a physical lifetime (Section 3.6).

Table S6. Largest relative deviation (%) from the finest common reference as a function of the momentum grid (32,768 bins) and of the number of transfer-energy bins (N = 384). "Seven quantities" refers to the rows of Table S5 at their original η; "η = 0.25 meV" is the same set recomputed with η = 0.25 meV and compared with the finest reference at that η.

Varied setting	Value	Seven quantities (original η)	Same quantities at η = 0.25 meV	$R(\lambda = 1)$
Grid $N \times N$	128 × 128	5.0×10^{-4}	5.0×10^{-4}	13.6×10^{-6}
Grid $N \times N$	192 × 192	6.7×10^{-4}	7.6×10^{-4}	5.1×10^{-6}
Grid $N \times N$	256 × 256	4.6×10^{-4}	4.6×10^{-4}	16.4×10^{-6}
Grid $N \times N$	384 × 384	1.7×10^{-4}	1.7×10^{-4}	8.0×10^{-6}
Grid $N \times N$	512 × 512	2.2×10^{-4}	2.2×10^{-4}	1.7×10^{-6}
Grid $N \times N$	768 × 768	1.0×10^{-4}	1.0×10^{-4}	0.1×10^{-6}
Grid $N \times N$	1024 × 1024	1.2×10^{-4}	1.2×10^{-4}	0.9×10^{-6}
Energy bins	8,192	1.09×10^{-3}	1.09×10^{-3}	41.9×10^{-6}
Energy bins	16,384	5.9×10^{-4}	5.9×10^{-4}	9.2×10^{-6}
Energy bins	65,536	2.0×10^{-4}	2.0×10^{-4}	5.0×10^{-6}
Energy bins	131,072	0.7×10^{-4}	1.0×10^{-4}	1.6×10^{-6}

S9. Continuum Edges and Peak Widths

This section documents the position of the plasmon peaks relative to the particle–hole continua of the model and the dependence of their widths on the broadening η. The edges were obtained from the bare density response of the four-band model at 300 K. The upper edge of the intraband continuum is the largest intraband transition energy at the given wave vector, as defined in the calculation code. The onset of the interband continuum is the lowest transition energy with nonzero occupation-weighted weight on the finite grid, with no additional amplitude cutoff. The edges depend weakly on the grid (the intraband edge at q = 0.2 nm^{-1} is 58.860, 58.860 and 58.861 meV for 384 × 384, 768 × 768 and 1024 × 1024 points), whereas the interband onset is sensitive to the sampling of the Brillouin zone (3.5, 2.9 and 2.4 meV for the same grids) because the occupation differences become small near the Fermi level at 300 K.

Table S7. Edges of the bare particle–hole continua and positions of the plasmon peaks (φ = 0°, T = 300 K, d = 2 nm, κ = 4; grid 1024 × 1024, 131,072 bins).

q (nm^{-1})	μ (eV)	Intraband upper edge (meV)	Interband onset (meV)	Peak	Energy (meV)	Energy / intraband edge
0.2	0	58.86	2.37	acoustic	187.37	3.18
0.2	0	58.86	2.37	optical	413.09	7.02
0.2	0	58.86	2.37	dynamic hBN, acoustic	214.00	3.64
0.2	0	58.86	2.37	dynamic hBN, optical	409.30	6.95
0.05	0	14.72	1.94	acoustic	50.18	3.41
0.05	0	14.72	1.94	optical	219.77	14.93
0.05	0.2	14.72	1.94	acoustic	42.55	2.89
0.05	0.2	14.72	1.94	optical	185.30	12.59

All peaks lie above the intraband continuum, by a factor of 2.9 or more. The interband continuum starts below all peaks, so the peaks are located inside the energy range of interband transitions, but the bare interband spectral weight at the peak energies is very small; its values in the energy bin that contains each peak and in a window of ±0.25 meV around it are listed in the deposited file R_T2_peak_continuum_comparison.csv.

The width of a peak is defined as the full width at half maximum (FWHM) of the unit-normalized charge loss, without background subtraction. For η from 2 to 0.05 meV the FWHM equals 2η to within 0.1% for the acoustic and optical modes at q = 0.05 nm^{-1} (μ = 0 and 0.2 eV) and for the acoustic mode at q = 0.2 nm^{-1}, with grid-to-grid differences between 768 × 768 and 1024 × 1024 points below 0.02%. The optical mode at q = 0.2 nm^{-1} shows an excess width that grows as η decreases, from 0.2% at η = 2 meV and 0.8% at 0.5 meV to 8% at 0.05 meV, and its width changes by up to 5.7% between the two grids at η = 0.05 meV, so that its low-η widths are not converged with respect to the grid. The damping rate obtained from the dielectric function, $\gamma = \mathrm{Im}\,\varepsilon/(\partial\,\mathrm{Re}\,\varepsilon/\partial E)$ evaluated at the peak, equals η for the same modes within the same accuracy (for example 0.05002 meV for η = 0.05 meV and the acoustic mode at q = 0.2 nm^{-1}), so that the quality factor $E_\mathrm{p}/(2\gamma)$ in this calculation is set by η and is not a physical lifetime.

Table S8. Peak widths of the plasmons ($\varphi = 0°$, $T = 300$ K, $d = 2$ nm, $\kappa = 4$). E_p and FWHM are the peak energy and the full width at half maximum of the unit-normalized charge loss at $\eta = 0.05$ meV (grid 1024 × 1024); the intercept is the linear extrapolation of the FWHM to $\eta \to 0$ from $\eta = 0.05$, 0.1 and 0.25 meV, with its standard error.

q (nm^{-1})	μ (eV)	Branch	E_p (meV)	FWHM at η = 0.05 meV (meV)	FWHM($\eta \to$ 0) (meV)	Standard error (meV)	Grid check (768 vs 1024)
0.2	0	acoustic	187.37	0.100053	5.65e-05	9.6e-06	passed
0.2	0	optical	413.09	0.108331	8.80e-03	8.2e-04	not passed
0.05	0	acoustic	50.18	0.100008	9.30e-06	9.1e-07	passed
0.05	0	optical	219.77	0.100037	2.90e-05	6.7e-06	passed
0.05	0.2	acoustic	42.55	0.100000	1.50e-06	6.9e-07	passed
0.05	0.2	optical	185.30	0.100000	1.10e-07	4.1e-08	passed

The extrapolated widths are smaller than 10^{-4} meV for all modes whose grid check is passed. For the optical mode at $q = 0.2$ nm^{-1} the extrapolation gives 8.8×10^{-3} meV, which is an upper bound for the intrinsic width in this calculation, as the grid check is not passed. Since the discrete momentum grid yields a discrete spectrum when η is taken to zero, these results characterize the discretized response and do not prove the absence of damping in the continuum limit.

S10. Layer Asymmetry from Unequal Filling and Broadening

Section 3.4 considers the layer asymmetry that arises from the dielectric environment. Here the structural environment is kept symmetric (gapless hBN, equal upper and lower dielectric constants) and the layers are made unequal electronically, either through different chemical potentials $\mu_1 = 0$ and $\mu_2 = \delta\mu$ or through different broadenings $\eta_1 = 0.5$ meV and η_2. The interlayer charge response is decomposed into layer-odd-like and layer-even-like branches by projecting the physical charge eigenvectors onto the layer-odd combination, and the branch correction is that of Equation (20); at the symmetric point the decomposition reduces to the one used in Section 3.4, and the two branches add up to the full RPA electronic correction. The layer-odd projection of the selected branch has a purity above 0.9997 in all cases of Table S9.

Table S9. Dark-branch ratio R of Equation (20) for unequal layer fillings and broadenings in the gapless symmetric hBN structure (q = 0.2 nm^{-1}, $\varphi = 0°$, T = 300 K, d = 2 nm, upper and lower dielectric constants 4; 384 × 384 grid, 32,768 bins; layer 1: $\mu_1 = 0$, η_1 = 0.5 meV; window 195–320 meV, energy step 0.25 meV). R is the ratio of the layer-odd-like electronic correction areas of the same-domain and the opposite-domain stack. Peak energies are the sampled maxima of the layer-odd and layer-even charge projections (step 0.25 meV).

Case	$\delta\mu = \mu_2 - \mu_1$ (meV)	η_2/η_1	R (%)	Layer-odd peak (meV)	Layer-even peak (meV)	Minimum layer-odd purity
Unequal filling	0	1	0	214.00	409.25	1.000000
Unequal filling	2	1	3.84×10^{-4}	214.00	409.00	1.000000
Unequal filling	5	1	2.36×10^{-3}	213.75	408.50	0.999998
Unequal filling	10	1	9.21×10^{-3}	213.25	407.75	0.999993
Unequal filling	20	1	3.52×10^{-2}	212.50	406.25	0.999972
Unequal filling	50	1	2.01×10^{-1}	210.25	402.00	0.999824
Unequal broadening	0	2	-5.33×10^{-5}	214.00	409.25	0.999999
Unequal broadening	0	5	-8.50×10^{-4}	214.00	409.25	0.999991
Unequal broadening	0	10	-4.28×10^{-3}	214.00	409.25	0.999954
Unequal broadening	0	20	-1.89×10^{-2}	214.25	409.25	0.999794
Both	10	5	7.31×10^{-3}	213.25	407.75	0.999985

For unequal fillings R grows with $\delta\mu$ as a power law $R \propto \delta\mu^p$ with p = 1.96 (fit over $\delta\mu$ = 2–20 meV) and 1.95 (2–50 meV). For unequal broadenings $R \propto (\eta_2/\eta_1 - 1)^p$ with p = 1.99 (η_2/η_1 = 2–20). The quadratic dependence is the one expected for a symmetry-breaking perturbation of first-order amplitude. With unequal broadenings the signed electronic correction of the branch can be negative, so that R is not an absorption ratio, and we report it with its sign. The largest value, 0.20% at $\delta\mu$ = 50 meV, is about ten times smaller than the 2.04% of the structural asymmetry at $|\lambda|$ = 1. Unequal filling shifts the layer-odd and layer-even peaks to lower energy, by 3.8 meV and 7.3 meV at $\delta\mu$ = 50 meV, whereas unequal broadening does not shift them beyond the sampling step.

S11. Electron Number, Rb-Vacancy Mapping and Moderate Density Changes

At $\mu = 0$ and 300 K the four-band model contains 2.988573 electrons per unit cell (a = 4.020 Å), of which the xy orbitals hold 1.919377 and the xz and yz orbitals 0.534598 each for the two spin-resolved blocks (the xy orbitals of the two blocks hold 0.959689 each). The corresponding areal density of the four retained bands is 1.849×10^{15} cm^{-2}; it contains the full occupation of the retained bands and is not the density of mobile carriers. The value μ = 0 is the energy reference of the fitted model, and Ref. 7 of the main text does not state the electron number of the stoichiometric compound in this model. We therefore do not identify $\mu = 0$ with $\delta = 0$ and use the relative mapping $n(\mu\delta) = n(0) - \delta$, with $\Delta n = -\delta/a^2$ per unit cell, in Table S10. The nominal reference of three electrons per cell differs from $n(0)$ by 0.0114 electrons per cell, equivalent to 7.1×10^{12} cm^{-2}.

Table S10. Response for changes of the electron density relative to μ = 0 (T = 300 K, φ = 0°, d = 2 nm, κ = 4; stiffness on a 512 × 512 grid; RPA at q = 0.05 nm^{-1} with a 256 × 256 grid and η = 1 meV, and at q = 0.2 nm^{-1} with a 384 × 384 grid and η = 0.5 meV, both with 32,768 bins). Rb-vacancy rows assume that each vacancy removes one electron and that the cell contains one formula unit; they are model scenarios and not calibrated compositions. Changes are relative to μ = 0.

Case	Δn (10^{13} cm^{-2})	μ (meV)	D_{xx} change (%)	D^s_{xx} change (%)	Eac, q = 0.05 nm^{-1} change (%)	Eac, q = 0.2 nm^{-1} change (%)	$\lvert\Pi_{SN}/\Pi_{NN}\rvert^2$, q = 0.05 nm^{-1} change (%)	$\lvert\Pi_{SN}/\Pi_{NN}\rvert^2$, q = 0.2 nm^{-1} change (%)
Rb vacancy, δ = 0.12	−7.43	−53.29	+9.55	−0.67	+4.47	+4.43	−17.36	−17.30
Rb vacancy, δ = 0.17	−10.52	−72.85	+13.62	−1.45	+6.31	+6.25	−24.20	−24.11
Rb vacancy, δ = 0.22	−13.61	−91.20	+17.54	−2.55	+8.05	+7.98	−30.60	−30.50
Holes removed	−1.00	−7.77	+1.24	+0.01	+0.59	+0.59	−2.35	−2.34
Holes removed	−3.00	−22.74	+3.76	−0.07	+1.79	+1.78	−7.06	−7.03
Holes removed	−5.00	−36.96	+6.36	−0.27	+3.00	+2.97	−11.76	−11.71
Holes removed	−10.00	−69.65	+12.94	−1.29	+6.00	+5.95	−23.08	−23.00
Electrons added	+1.00	7.95	−1.23	−0.04	−0.59	−0.59	+2.34	+2.33
Electrons added	+3.00	24.34	−3.67	−0.25	−1.79	−1.77	+7.02	+6.99
Electrons added	+5.00	41.29	−6.15	−0.62	−3.01	−2.99	+11.77	+11.71
Electrons added	+10.00	85.43	−12.74	−2.37	−6.36	−6.32	+24.41	+24.29

Rb vacancies in this mapping move the system to the hole side of the scan of Figure 5. The stiffness and the acoustic energy increase, the relative spin amplitude decreases, and the effects are close to linear in Δn up to 10^{14} cm^{-2}. The changes for electron and hole doping of equal size differ by less than 0.4 percentage points in the energy and by up to 1.3 percentage points in the relative spin amplitude (10^{14} cm^{-2}), reflecting a weak curvature of the dependence.

S12. Fermi-Surface Topology and Temperature Dependence of the Softening

The bands and the periodic Fermi contours were evaluated on a 2001 × 2001 grid for μ = 0–0.3 eV in steps of 5 meV; the stiffness was evaluated on a 1024 × 1024 grid for μ = −0.1 to 0.35 eV in steps of 5 meV at T = 300, 100 and 30 K, with only the Fermi function changed. The xy-type pockets at the X and Y points (($k_x a/\pi$, $k_y a/\pi$) = (0, ±1) and (±1, 0)) have their band maximum, with xy orbital weight 1, at μL = 175.2235 meV. Between 175 and 180 meV the number of periodic Fermi contours decreases from three to two. A second xy-type pocket has its band maximum at −27.256 meV and is already absent at μ = 0. Stationary points, contour numbers and pocket areas at all μ are listed in the deposited files R_T5_stationary_points.csv, R_T5_topology.csv and R_T5_pocket_areas.csv.

Table S11. Slope of the long-wavelength acoustic plasmon energy at $\mu = -0.1$ eV and position of the local curvature extremum (5 meV steps, $q = 0.05\ \mathrm{nm}^{-1}$ long-wavelength estimate).

T (K)	kBT (meV)	dELW/dμ at $\mu = -0.1$ eV (meV/eV)	Curvature extremum near μL (meV)
300	25.85	−48.37	175.0
100	8.62	−49.32	175.0
30	2.59	−49.08	175.0

The slope is negative at the left end of the scan at all three temperatures, so that the softening does not start at μL. The curvature extremum lies at the grid point nearest to μL and is a diagnostic from 5 meV differences; at 300 K the thermal width smooths the structure near μL. At 30 K the stiffness on the 512 $\times$ 512 grid deviates from that on the 1024 $\times$ 1024 grid by up to 0.21%, so the second differences at 30 K are not converged and are used only as an indication.

S13. Relative Twist of the Two Layers

The second layer is rotated by θ about the z axis. Only the Coulomb interaction couples the layers, so the bare response of the second layer is evaluated at the wave vector rotated into its crystal frame, $\mathbf{q}' = R(-\theta)\mathbf{q}$, while the operators N and S, the spin axis and the Coulomb kernel are unchanged; the second layer then propagates along $\varphi - \theta$ in its own frame. Two internal checks were passed: $\theta = 0$ reproduces the aligned result, and the same-domain stack at $\theta = 90°$ equals the opposite-domain stack at $\theta = 0$, with a largest relative difference of 1.2×10^{-13} over energies and areas ($\varphi = 0°$ and $22.5°$). The areas follow the convention $W = (1/\pi)\int(-\mathrm{Im}\,\chi)\,\mathrm{d}E$ of the main-text tables; the raw integrals without the factor $1/\pi$ are kept in the deposited file R_T6_peak_areas.csv.

https://doi.org/10.3390/nano16211430

Table S12. Dependence on the relative twist θ of the second layer (q = 0.2 nm^{-1}, μ = 0, T = 300 K, η = 0.5 meV, d = 2 nm, κ = 4; 384 × 384 grid, 32,768 bins). Dark/bright ratios are the total-spin areas in the dark domain configuration divided by those in the bright configuration at the same θ and φ: same-domain over opposite-domain for the acoustic plasmon, opposite-domain over same-domain for the optical plasmon. Areas are $W = (1/\pi)\int(-\mathrm{Im}\,\chi)\,\mathrm{d}E$ over ±12 meV around the real-axis crossing, without background subtraction. For θ > 45° the roles of dark and bright branches are exchanged.

φ (°)	θ (°)	Eac (meV)	Eopt (meV)	Acoustic dark/bright (%)	Optical dark/bright (%)
0	0	187.36	413.09	0.038	0.0523
0	2.5	187.36	413.09	0.0386	0.0528
0	5	187.34	413.08	0.0447	0.0589
0	10	187.28	413.06	0.133	0.152
0	15	187.18	413.02	0.531	0.576
0	22.5	187.00	412.94	2.85	3.02
0	30	186.82	412.87	10.8	11.2
0	45	186.63	412.79	100	100
0	60	186.82	412.87	929	892
0	75	187.18	413.02	1.9×10^4	1.7×10^4
0	90	187.36	413.09	2.6×10^5	1.9×10^5
22.5	0	186.64	412.79	0.12	0.101
22.5	2.5	186.70	412.82	0.26	0.254
22.5	5	186.76	412.85	0.61	0.627
22.5	10	186.87	412.89	1.53	1.6
22.5	15	186.95	412.92	2.34	2.47
22.5	22.5	187.00	412.94	2.85	3.02
22.5	30	186.95	412.92	2.34	2.47
22.5	45	186.64	412.79	0.12	0.101
22.5	60	186.32	412.66	21.5	21.7
22.5	75	186.32	412.66	466	460
22.5	90	186.64	412.79	8.4×10^4	9.9×10^4

At φ = 0° the dark/bright ratio grows from 0.04–0.05% at θ = 0 to 0.13–0.15% at 10°, 0.5–0.6% at 15°, about 3% at 22.5°, about 11% at 30° and 100% at 45°, and the roles are exchanged for larger θ. The projection estimate $[\cos 2\varphi \mp \cos 2(\varphi - \theta)]^2$ for the weight of the branches, whose dark/bright ratio at φ = 0° is $\tan^4\theta$ (0.097% at 10°), reproduces the trend; the full spectra contain additional background. The peaks move by up to 0.7 meV (acoustic) and 0.3 meV (optical) at θ = 45° at φ = 0°.

S14. Sensitivity to the Tight-Binding Parameters

The following modifications keep the symmetry relation $H(k_x, k_y) = U\,H(k_y, k_x)\,U^\dagger$ of Section 2.3: all hoppings multiplied by 0.8, 0.9, 1.1 and 1.2 (on-site energies unchanged); the two spin–orbit parameters multiplied by 0, 0.5, 2 and 10; the on-site energy of the xy orbital shifted by ±50 and ±100 meV; and ten random samples in which each of the ten independent hopping parameters is multiplied by a uniform factor in [0.9, 1.1] (seed 20261030), the rotation-related matrix elements keeping their relations to the original parameters. The covariance of the Hamiltonian holds to 1.2×10^{-15} eV in all 23 sets including the original one. At φ = 45° the mixed-vertex amplitudes r and c vanish to within 5.5×10^{-15} in all sets.

https://doi.org/10.3390/nano16211430

Table S13. Sensitivity to symmetry-preserving parameter changes (q = 0.2 nm^{-1}, φ = 0°, T = 300 K, η = 0.5 meV, d = 2 nm, κ = 4; response on a 384 × 384 grid with 32,768 bins, stiffness on 512 × 512). Energies and P^2 are for μ = 0. Softening is the decrease of the acoustic energy between the lower and the upper end, at fixed μ (0 → 0.2 eV) and at fixed electron number (the electron numbers of the original parameters at μ = 0 and 0.2 eV). The last column is the largest ratio of the same-domain to the opposite-domain acoustic total-spin area (full spectra including background) of the two conditions.

Setting	Eac (meV)	Eopt (meV)	P^2	Softening, fixed μ (%)	Softening, fixed n (%)	Same/opposite acoustic area
Original parameters	187.4	413.1	0.566	15.1	15.1	3.8 × 10^{-4}
Hoppings × 0.8	151.6	334.2	0.708	11.2	10.0	4.5 × 10^{-4}
Hoppings × 0.9	169.8	374.5	0.625	13.4	13.1	4.1 × 10^{-4}
Hoppings × 1.1	205.2	452.3	0.513	14.9	16.1	3.9 × 10^{-4}
Hoppings × 1.2	223.2	492.0	0.465	14.1	16.5	3.9 × 10^{-4}
SOC × 0	187.4	413.1	0.566	15.1	15.1	3.8 × 10^{-4}
SOC × 0.5	187.4	413.1	0.566	15.1	15.1	3.8 × 10^{-4}
SOC × 2	187.4	413.1	0.566	15.1	15.1	3.9 × 10^{-4}
SOC × 10	187.1	412.1	0.561	15.0	15.0	5.9 × 10^{-4}
xy on-site shift (eV) −0.1	174.2	382.6	0.666	9.4	10.8	3.5 × 10^{-4}
xy on-site shift (eV) −0.05	180.6	397.6	0.613	12.5	13.2	3.7 × 10^{-4}
xy on-site shift (eV) +0.05	195.6	432.1	0.512	16.8	16.2	4.3 × 10^{-4}
xy on-site shift (eV) +0.1	205.2	454.0	0.456	17.7	16.7	5.2 × 10^{-4}
Random sample 01	186.2	410.7	0.552	15.2	15.2	4.1 × 10^{-4}
Random sample 02	174.1	383.9	0.612	13.5	13.3	4.1 × 10^{-4}
Random sample 03	189.2	417.2	0.505	15.9	15.3	4.7 × 10^{-4}
Random sample 04	192.8	425.6	0.524	16.3	16.3	4.2 × 10^{-4}
Random sample 05	198.6	438.5	0.527	16.7	16.7	3.9 × 10^{-4}
Random sample 06	186.9	412.5	0.526	16.4	15.5	4.4 × 10^{-4}
Random sample 07	175.8	387.7	0.583	15.3	14.3	4.2 × 10^{-4}
Random sample 08	182.4	402.6	0.493	16.8	15.5	5.3 × 10^{-4}
Random sample 09	188.1	415.5	0.510	17.7	16.2	4.7 × 10^{-4}
Random sample 10	198.5	437.6	0.545	15.7	16.2	3.7 × 10^{-4}

The ratio of the same-domain to the opposite-domain acoustic total-spin area, computed from the full spectra, is below 6 × 10^{-4} in all 92 evaluated rows, so that the selection is retained. This ratio includes the bare spin background and is not the residue-based selection rule itself. The energies, P^2 and the amount of softening depend on the parameters, whereas the direction of the softening is the same in all sets. A rescaling of the hoppings changes the energies approximately in proportion, a shift of the xy on-site energy by ±0.1 eV changes P^2 between 0.46 and 0.67, and the spin–orbit coupling has little effect on the charge plasmons in this model (the acoustic energy changes by 0.15% for a tenfold increase).

S15. Absolute Spin and Charge Spectral Weights

The scan covered μ = −0.2 to 0.2 eV in steps of 50 meV, with additional points at 0.025, 0.075, 0.125 and 0.175 eV, at q = 0.05 and 0.2 nm^{-1} (52 rows in the deposited file

R_T8_absolute_weights.csv). In all four cases the spin weight is largest at the upper end of the scan, μ = 0.2 eV.

Table S14. Integrated spectral weights of the spin and charge peaks between μ = 0 and 0.2 eV ($\varphi = 0°$, T = 300 K, η = 0.5 meV, d = 2 nm, κ = 4; 384 × 384 grid, 32,768 bins). $W = (1/\pi) \int (-\mathrm{Im}\,\chi)\, \mathrm{d}E$ over ±5 full widths at half maximum around the respective peak, in $Å^{-2}$, without background subtraction. The spin probe is $S_1 + S_2$ with $S = 2S_z/\hbar$; the opposite-domain acoustic plasmon is paired with the density difference $N_1 - N_2$ and the same-domain optical plasmon with $N_1 + N_2$. WS/WN is the ratio of the spin to the charge weight in their own windows.

q (nm^{-1})	Plasmon	WS, μ = 0	WS, μ = 0.2 eV	WS change (%)	WN change (%)	WS/WN, μ = 0 → 0.2 eV	Squared bare vertex ratio, μ = 0 → 0.2 eV
0.05	acoustic	6.04×10^{-5}	7.90×10^{-5}	+30.7	−16.2	0.579 → 0.904	0.578 → 0.904
0.05	optical	1.35×10^{-5}	1.80×10^{-5}	+33.6	−15.7	0.567 → 0.899	0.567 → 0.899
0.2	acoustic	2.59×10^{-4}	3.38×10^{-4}	+30.5	−16.3	0.580 → 0.904	0.580 → 0.904
0.2	optical	1.15×10^{-4}	1.54×10^{-4}	+33.1	−15.8	0.569 → 0.900	0.569 → 0.900

The spin weight increases by 30.5–33.6% between μ = 0 and 0.2 eV, whereas the charge weight decreases by 15.7–16.3%, so that the ratio increases by 56–59%, in agreement with the squared bare vertex ratio to within 2.2% over the scan for a common window. The values are areas of the model spectra at the numerical broadening η = 0.5 meV and are not calibrated experimental counts.

S16. Layer-Weighted Detection

The layer amplitudes s_i are those of Figure 3, evaluated at the real-axis crossing and normalized to δN_1 = 1 and $\delta N_2 = \mp 1$ for the acoustic and optical plasmon; they are not complex-frequency eigenvectors. For the same-domain acoustic plasmon $s_2 = -s_1$, so that the weighted signal is $(1 - e^{-qd})s_1$, whereas for equally phased layer spins it is $(1 + e^{-qd})s_1$; the intensity ratio is $\tanh^2(qd/2)$. The two-sided readings give the matrix A = [[1, e^{-qd}], [e^{-qd}, 1]], whose 2-norm condition number is $(1 + e^{-qd})/(1 - e^{-qd})$. The table gives representative values for q = 0.05, 0.1 and 0.2 nm^{-1}; the values for q = 0.05–0.2 nm^{-1} in steps of 0.025 nm^{-1} are in the deposited files R_T9_weighted_signals.csv and R_T9_weight_error_corners.csv.

Table S15. Layer-weighted detection with the weights $(1, e^{-qd})$ ($\mu = 0$, $\varphi = 0°$, $T = 300$ K, $\eta = 0.5$ meV, $\kappa = 4$; 384 × 384 grid, 32,768 bins). Same-domain acoustic leakage is the intensity of the weighted spin signal of the same-domain acoustic plasmon relative to that of equally phased layer spins, $\tanh^2(qd/2)$. The condition number is that of the matrix of two readings with weights $(1, e^{-qd})$ and $(e^{-qd}, 1)$. Worst-case layer-difference errors are for independent ±5% errors of the four weights (0.05 times the condition number); for a calibration error of each layer that is the same in both readings the error is 5%.

q (nm^{-1})	d (nm)	e^{-qd}	Same-domain acoustic leakage (%)	Condition number	Worst-case layer-difference error, independent weights (%)
0.050	1	0.951	0.06	40.0	200
0.050	2	0.905	0.25	20.0	100
0.050	5	0.779	1.55	8.0	40
0.100	1	0.905	0.25	20.0	100
0.100	2	0.819	0.99	10.0	50
0.100	5	0.607	6.00	4.1	20
0.200	1	0.819	0.99	10.0	50
0.200	2	0.670	3.90	5.1	25
0.200	5	0.368	21.36	2.2	11

The results do not include experimental noise, spatial inhomogeneity or the unknown coupling of a real probe to the spin.

References

20. Gunnink, P.M.; Sinova, J.; Mook, A. Spin demons in d-wave altermagnets. *Phys. Rev. Lett.* **2025**, *135*, 126701. https://doi.org/10.1103/99zp-s88m
21. Rostami, H.; Hofmann, J. Fermi liquid theory of d-wave altermagnets: Demon modes and Fano-demon states. *Phys. Rev. Lett.* **2025**, *135*, 236701. https://doi.org/10.1103/zqz6-sq2n

Disclaimer/Publisher's Note: The statements, opinions and data contained in all publications are solely those of the individual author(s) and contributor(s) and not of MDPI and/or the editor(s). MDPI and/or the editor(s) disclaim responsibility for any injury to people or property resulting from any ideas, methods, instructions or products referred to in the content.
